# Understorey light quality affects leaf pigments and leaf phenology in different plant functional types

**DOI:** 10.1111/ppl.13723

**Published:** 2022-06-14

**Authors:** Craig C. Brelsford, Marieke Trasser, Tom Paris, Saara M. Hartikainen, T. Matthew Robson

**Affiliations:** ^1^ Yield Systems Espoo Finland; ^2^ Organismal and Evolutionary Biology (OEB), Viikki Plant Science Centre (ViPS), Faculty of Biological and Environmental Sciences University of Helsinki Helsinki Finland; ^3^ Gregor Mendel Institute of Molecular Plant Biology Vienna Austria; ^4^ Vienna BioCenter PhD Program Doctoral School of the University of Vienna and Medical University of Vienna Vienna Austria; ^5^ Ecodiv Normandie Université, UNIROUEN Rouen France

## Abstract

Forest understorey plants receive most sunlight in springtime before canopy closure, and in autumn following leaf‐fall. We hypothesised that plant species must adjust their phenological and photoprotective strategies in response to large changes in the spectral composition of the sunlight they receive. Here, we identified how plant species growing in northern deciduous and evergreen forest understoreys differ in their response to blue light and ultraviolet (UV) radiation according to their functional strategy. We installed filters in a forest understorey in southern Finland, to create the following treatments attenuating: UV radiation below 350 nm, all UV radiation (< 400 nm), all blue light and UV radiation (< 500 nm), and a transparent control. In eight species, representing different functional strategies, we assessed leaf optical properties, phenology, and epidermal flavonoid contents over two years. Blue light accelerated leaf senescence in all species measured in the understorey, apart from *Quercus robur* seedlings, whereas UV radiation only accelerated leaf senescence in *Acer platanoides* seedlings. More light‐demanding species accumulated flavonols in response to seasonal changes in light quality compared to shade‐tolerant and wintergreen species and were particularly responsive to blue light. Reduction of blue and UV radiation under shade reveals an important role for microclimatic effects on autumn phenology and leaf photoprotection. An extension of canopy cover under climate change, and its associated suppression of understorey blue light and UV radiation, may delay leaf senescence for understorey species with an autumn niche.

## 1 INTRODUCTION

Forest understories are dynamic and heterogeneous light environments. Many plant species exploit increasing sunlight reaching the understorey prior to canopy closure for photosynthesis during early spring, while some also extend their growing season to capitalise on favourable conditions after canopy leaf senescence in the autumn (Kudo et al., 2008; Richardson & O'Keefe, 2009). Different plant species segregate into functional types with differing light‐capture strategies within the forest understorey plant community (Grubb, 1977; Heberling et al., 2019; Wolkovich & Cleland, 2011). Accordingly, species may adopt different strategies when timing their growth and life cycle, adapting to seasonal variation in light availability in the forest understorey (Augspurger & Salk, 2017; Heberling et al., 2019). These seasonal adaptations partially drive differences in species composition beneath deciduous and evergreen canopies (Frelich et al., 2003).

Environmental cues help plants to appropriately time their phenology (Flynn & Wolkovich, 2018; Chuine & Régnière, 2017). Spring ephemeral species emerge and leaf out to maximise carbon gain in spring, then senesce when the irradiance drops during canopy closure in deciduous stands (Kudo et al., 2008). Canopy leaf senescence and leaf‐fall in autumn once again make more sunlight available to the forest understorey (Richardson & O'Keefe, 2009). This autumn light niche is exploited by many perennial herbaceous species and tree seedlings, which delay leaf senescence until or beyond canopy opening, extending photosynthesis for overwinter carbon storage (Kudo et al., 2008; Augspurger et al., 2005). Wintergreen plants produce new leaves each year which overwinter and then senesce during the following growing season (Heberling et al., 2019). Although carbon gain is often negligible during winter, this strategy allows them to exploit favourable conditions for photosynthesis in early spring during snowmelt (Saarinen et al., 2016; Landhäusser et al., 1997). Shade‐tolerant species in the understorey may emerge later but extend their leaf longevity by utilising low light levels efficiently, whereas the leaves of facultative light‐exploiting or relatively light‐demanding species often emerge earlier to capitalise on relatively high understorey irradiances before canopy closure (Niinemets, 2010).

Once the canopy closes, the understorey light environment is mostly shade (Chazdon & Pearcy, 1991). Not only does the amount of irradiance change in the understorey, but so does its spectral composition, or light quality (Hartikainen et al., 2018; Leuchner et al., 2005; Messier et al., 1998; Ross and Flanagan, 1986; Federer & Tanner, 1966; Vezina & Boulter, 1966). Leaves in the canopy reflect or transmit most far‐red (FR) light, which is scattered into the understorey shade, reducing the ratio of red to far red (R:FR) light (Ballaré & Pierik, 2017). These changes in R:FR in the understorey are detected by phytochrome photoreceptors, which coordinate the shade avoidance syndrome in many species (Ballaré et al., 1987). Similarly, the irradiance of blue light penetrating into the forest understorey is reduced during canopy leaf out, due to its absorption by the plant canopy (Casal, 2013). Although UV radiation is also reduced, the ratio of UV:PAR (photosynthetically active radiation) increases in understorey shade due to the higher diffusivity of UV radiation (Flint & Caldwell, 1998; Grant et al., 2005).

Plants produce photoprotective pigments such as carotenoids and flavonoids in response to high‐light stress and UV radiation (Agati et al., 2020; Agati & Tattini, 2010). There is increasing evidence that flavonoid accumulation is mediated through cryptochromes (CRYs) in response to blue light and down to 350‐nm wavelength of UV‐A radiation, while UV Resistance Locus 8 (UVR8) largely dictates plant responses to UV‐B radiation and up to 350‐nm wavelength of UV‐A radiation (Brelsford, Morales, et al., 2019a; Rai et al., 2019; Rai et al., 2020). The flavonoids in plant leaves partly function as antioxidants but can be broadly separated into flavonols/flavones which also screen UV‐radiation, and anthocyanins which absorb blue‐green light, and to a lesser‐extent UV radiation (Agati & Tattini, 2010).

We know relatively little about how the role of flavonoids differs among plant functional types in response to light. It has been suggested that epidermal UV‐screening is highest in evergreen plants, intermediate in deciduous woody plants, and lowest in herbaceous plants (Day, 1993; Day et al., 1992; Li et al., 2010; Semerdjieva et al., 2003). Spectral attenuation in a common‐garden of 23 forb species, led to higher plasticity of flavonoid accumulation in response to UV‐B radiation among shade‐tolerant than shade‐intolerant species, while the opposite effect of light‐capture strategy was found for UV‐A radiation (Wang et al., 2020). Low UV screening, allowing transmission of UV >350 nm to the mesophyll may aid photosynthesis in low‐light environments (Turnbull et al., 2013). Seasonal trends in leaf flavonoids in the understorey plant community have been found to correlate with received irradiance, in particular UV‐A irradiance, which peaks in spring and autumn when the canopy is open (Hartikainen et al., 2020). While the patterns of received solar irradiance may govern the time‐course trends in epidermal flavonols, various environmental cues may account for the seasonal dynamics of anthocyanins in plant canopies, including temperature, herbivory and light stress (Archetti et al., 2009; Hoch et al., 2003; Karageorgou & Manetas, 2006; Lee, 2002). Nevertheless, anthocyanins typically increase in senescing leaves across many species (Feild et al., 2001; Lee, 2002). In woody plants, shading can reduce the production of anthocyanins during autumn, as well as delay leaf senescence, irrespective of the R:FR ratio (Lee et al., 2003). To date, it is still unknown what light signal, if any, is responsible for the reduction of autumnal anthocyanins and delayed leaf senescence under shaded conditions. Furthermore, climate change is expected to increase canopy duration, with earlier canopy leaf out and later leaf senescence (Buitenwerf et al., 2015; Piao et al., 2019). However, it is not clear how the changes in light quality under a prolonged period of canopy shading will affect the leaf pigments and phenology of understorey plants.

Here, we examine how solar blue light and UV radiation affect the seasonal dynamics of leaf pigments and phenology of plant species growing in forest understoreys, followed over a 2‐year period under filter treatments that selectively attenuated regions of the solar spectrum. We tested four hypotheses:

1. Based on our knowledge of flavonoid responses to seasonal changes in understorey light quality, attenuating blue light should cause the greatest reduction in flavonoid accumulation.

2. Flavonoid accumulation in relatively light‐demanding species should be more responsive than shade‐tolerant species in the understorey to blue and UV attenuation treatments, because they rely on periods of seasonally high irradiance, requiring rapid physiological adjustments in their photoprotection.

3. Changes in light quality during spring should have a greater effect on the phenology of tree seedlings than deciduous herbaceous species in the understorey. This is because the buds of tree seedlings are exposed to the sunlight above‐ground during winter and spring when deciduous herbaceous species are largely dormant below‐ground.

4. Attenuating UV radiation should reduce photodamage to leaves accumulated through the season and thus delay autumn leaf senescence.

## 2 MATERIALS AND METHODS

### 2.1 Climate and site information

Our experiment was conducted at the Lammi Biological Station, situated at 61.05°N, 25.05°E in Finland. The average mean monthly temperature and annual precipitation between 2017 and 2018 were 5.1°C and 572.4 mm, respectively. Daily mean, minimum and maximum temperatures (Figure S1), precipitation (Figure S2), and solar UV radiation and photosynthetically active radiation (PAR, Figures S3 and S4) were recorded at the site and processed by the Finnish Meteorological Institute.

The effect of canopy closure on the understorey spectral composition in the stands was measured in 2015 (Hartikainen et al., 2018), and during canopy leaf out between April and June in 2016, 2017 and 2018 (Table S1). Spectral irradiance was recorded with a Maya 2000 Pro array spectrometer (Ocean Optics) and calibrated for accuracy in the solar UV and PAR regions of the spectrum. The same device and protocol as Hartikainen et al. (2018) were used for measurements and processing of the irradiance data. Ambient solar PAR above the canopy at the Lammi Biological Station between 2015 and 2019 was also recorded to show the effects of changing cloudiness on incoming irradiance (PQS1 PAR Quantum Sensor, Kipp & Zonen; Figure S4).

### 2.2 Experimental design

Our 15 experimental plots were installed under two stand types: nine plots in deciduous stands dominated by either *Quercus robur* or *Betula pendula*, and six plots in an evergreen stand of *Picea abies*. Our plots were adjacent to those used by Hartikainen et al. (2018), with the same canopy species, stand spacing, architecture and LAI (Table S2).

**FIGURE 1:**
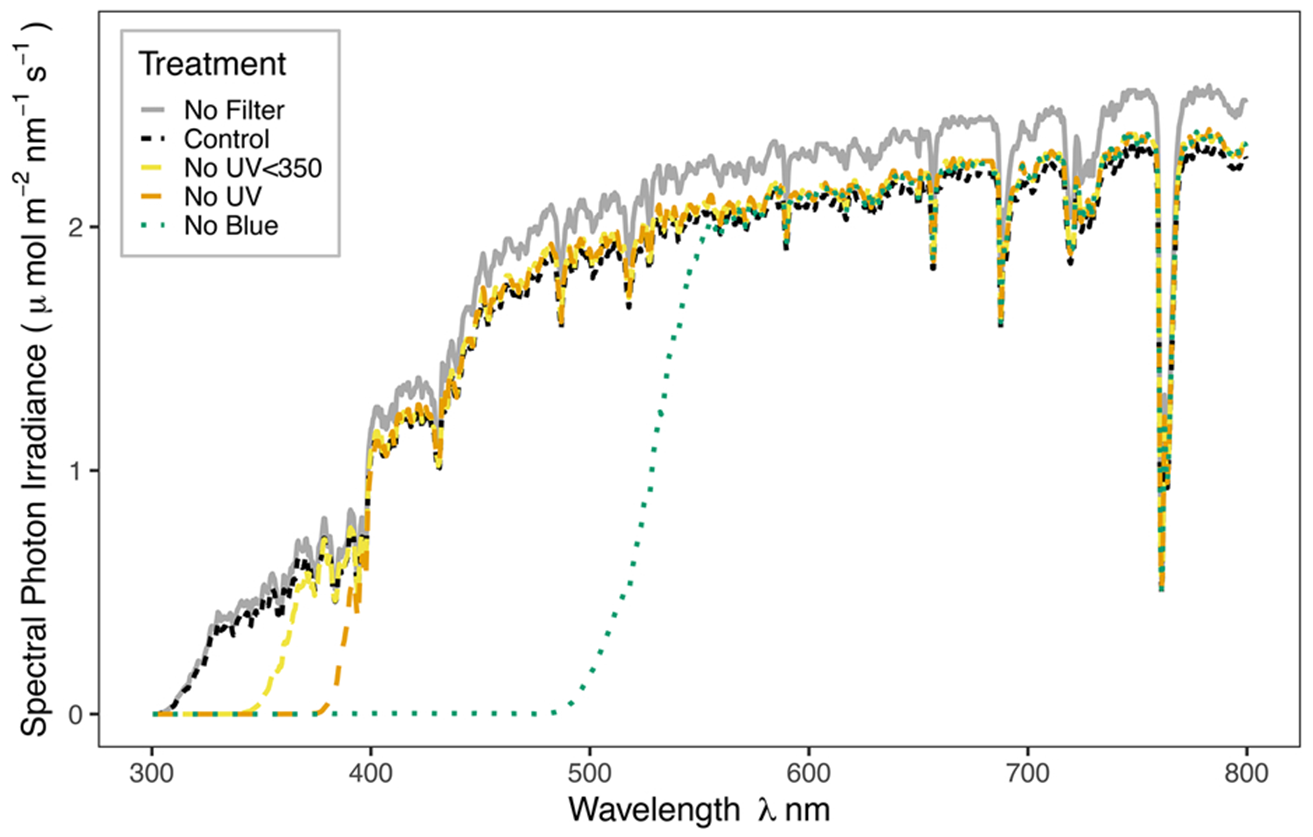
Understorey spectral irradiance in shade during spring leaf out on May 22, 2015. The measurements were taken in a deciduous *B. pendula* stand, following the methods described in Hartikainen et al. (2018). Spectral irradiance measurements under the forest stands between 2015 and 2018 are shown in Table S1.

We created a split‐plot design with one replicate from each of our four different polycarbonate filter treatments, randomly arranged within every plot. The control filter was equally transparent to all wavelengths of solar radiation (Plexiglas 2458 GT, Foiltek Oy), one filter treatment attenuated UV radiation below 350 nm (Plexiglas 0Z023, Foiltek Oy), one filter treatment attenuated all UV radiation (Arla Makrolife, Foiltek Oy), and one filter treatment attenuated blue light and all UV radiation (Plexiglas 1C33 (303), Foiltek Oy). Figure 1 gives the spectral transmittance of each filter in the understorey plots at solar noon in spring prior to canopy closure. The filter dimensions were 88 cm length × 60 cm width × 40 cm height. All filters were orientated with the longest‐sloping side facing south, and short vertical side facing north, with an air vent at the apex to increase air flow and reduce warming (Figure S5). Each filter was also raised on 10‐cm wooden blocks to allow airflow. We allowed a 10‐cm border around a central area inside the filters where plants were measured, to avoid filter‐edge effects (Aphalo et al., 2012).

### 2.3 Selection of understorey species

To capture responses from different plant functional types in the forest understorey, we used volunteer plant species already growing beneath the installed filters. We chose species with at least four separate plants underneath each filter (Table 1). These species were: *Aegopodium podagraria*, *Anemone nemorosa*, *Fragaria vesca*, *Maianthemum bifolium*, *Oxalis acetosella* and *Ranunculus cassubicus*. For these volunteer species, *R. cassubicus* and *A. podagararia* were only present in deciduous stands, and *M. bifolium* was only present in the evergreen stand. We also transplanted tree seedlings from two target species, *Acer platanoides* and *Quercus robur*, under the filters. Germinating seedlings were transplanted within the same stand at the two‐cotyledon stage, *A. platanoides* in April 2016 and *Q. robur* in September 2017. Seedlings of similar size were transplanted into those areas under the filters with the least existing vegetation to avoid disturbance and shading. Four *A. platanoides* seedlings were transplanted under each filter in both stand types, and four *Q. robur* seedlings were transplanted under each filter in the deciduous stands. *Q. robur* seedlings were not present in the evergreen stand and so were not transplanted there.

**TABLE 1:** Break‐down of plant species, samples and measurements.

Species	Functional type	Light capturestrategy	Phenological strategy	Stand type	Present under *n* filters	Present under *n* filters per treatment	Pigment measurements	Phenology measurements
*A. platanoides*	Tree seedling	More light‐demanding	Autumn senescing	Deciduous and evergreen	59	14.75	2017 and 2018	Spring and autumn 2017 and 2018
*Q. robur*	Tree seedling	More light‐demanding	Autumn senescing	Deciduous	23	5.75	2018	Autumn 2018
*A. nemorosa*	Herbaceous	More light‐demanding	Spring ephemeral	Deciduous and evergreen	49	12.25	2017	Spring 2018
*A. podagraria*	Herbaceous	More light‐demanding	Autumn senescing	Deciduous	25	6.25	2017 and 2018	Spring and autumn 2018
*R. cassubicus*	Herbaceous	More light‐demanding	Autumn senescing	Deciduous	20	5.00	2018	Spring and autumn 2018
*F. vesca*	Herbaceous	Shade tolerant	Wintergreen/ heterotypic	Deciduous and evergreen	52	13.00	2017 and 2018	
*M. bifolium*	Herbaceous	Shade tolerant	Summer green	Deciduous and evergreen	22	5.50	2017 and 2018	
*O. acetosella*	Herbaceous	Shade tolerant	Wintergreen	Deciduous and evergreen	41	10.25	2017 and 2018	

Our filters partially blocked precipitation from reaching the ground, so additional watering was provided to the plants every three days (Figure S6). The soil moisture in the plot was monitored with a 15‐cm TDR probe (SM200 Moisture Sensor with HH2 Moisture Meter, Delta‐T Devices), and we ensured soil moisture under the filters was equivalent to that outside and similar among treatments (Figure S6). Air temperature at 10‐ to 15‐cm height above the ground, monitored with iButton sensors (Maxim Integrated), was on average 0.3°C higher under the filters than in the ambient understorey (Figures S7 and S8), with no difference between treatments or filter types (Figure S7).

### 2.4 Measurements of leaf pigments and leaf phenology

A Dualex Scientific+ (Force‐A, University Paris‐Sud) was used to make nondestructive measurements of leaf pigments (chlorophyll content, epidermal flavonols and anthocyanins) based on their optical properties (Cerovic et al., 2012). Both adaxial and abaxial measurements of flavonols and anthocyanins were made every week between May 15 and October 2, 2017, and on five occasions between May 14 and October 1, 2018. For plants with few leaves, namely *A. platanoides*, *A. nemorosa*, *Q. robur* and *M. bifolium*, pigments were measured from all the leaves on each plant. For *A. podgraria*, *F. vesca* and *O. acetosella*, two leaves per plant were measured. Monitoring pigments by gently making repeated nondestructive Dualex measurements, enabled us to measure the same cohort of leaves through the growing season, from their production in spring until the last measuring dates in October 2017 and 2018, respectively, at which point the proportion of leaves which had senesced was too high for continued measurements. The relationships between the Dualex data and spectrophotometric readings of pigment absorbance from leaf extracts for the understorey species growing in these stands has been assessed by Hartikainen et al. (2020), but for our purposes the trends in epidermal optical properties attributable to flavonoids are of more interest, having greater functional relevance.

Bud burst and leaf out of *A. platanoides* was measured every 2–3 days, along a scale of 1–7 adapted from Teissier du Crois et al. (1981); whereby 1 = dormant, 2 = bud swelling, 3 = bud split, 4 = leaf tip protruding, 5 = leaf mostly out, 6 = leaf out but not fully expanded and 7 = fully expanded leaves. For the herbaceous species, *A. podagraria*, *A. nemorosa* and *R. cassubicus*, emergence of new leaves was measured once a week as 1 = shoot visible, and 2 = expanded leaf. Leaf senescence for all tree and herbaceous species was measured on a scale of 1–5, whereby 1 = fully green, 2 = starting to yellow, 3 = mostly yellow, 4 = turning brown and 5 = all leaves fallen. Leaf senescence was measured for all the leaves on each plant every two weeks. Bud burst was not recorded in *Q. robur*, which only germinated in spring 2018. The timing of canopy opening and canopy closure were recorded in the deciduous stands in both years, and determined as the date when the canopy had reached 50% leaf out (canopy closure) and 50% leaf fall (canopy opening).

A summary of species traits, phenology and phenological strategy is given in Table 1—time constraints limited pigment and phenological measurements of some species to just one year.

### 2.5 Statistical methods

The unit of replication for all statistical analyses was the filter treatment per plot. Model selection was based on meeting the requirements for statistical analyses laid out by Zuur et al. (2010). A linear mixed‐effects (LME) model was used to test the effects of Treatment × Stand type × Time as fixed factors, and with a nested random‐effects structure of Year/Time/Stand type/Stand/Plot ID, using the package ‘nlme’ (Pinheiro et al., 2017). When there were non‐linear trends over time, producing heteroscedasticity in residuals, a mixed effects generalised additive model (GAMM; package = ‘mgcv’, Wood & Wood, 2015), was used with Treatment × Stand Type as parametric terms, and a smoothing term for time, and for the random effects structure (Year/Time/Stand type/Stand/Plot ID) using the function bs = ‘re’ as described by (Pedersen et al., 2019). The spline used as the smoothing term for time was chosen based upon the model which had the lowest AIC value. If heteroscedasticity in the residuals was present due to heterogeneous variation, then the weighting function ‘weights = varPower’ was used. If filter treatment was significant in the model, then pairs of filters were compared to determine the effect of each spectral region: i.e. the results under the filter attenuating both blue light and UV were compared against those under the filter attenuating UV, to determine the effect of blue light. Similarly, responses in the filter treatment attenuating all UV were compared against those in the filter treatment attenuating UV below 350 nm, to determine the effect of UV radiation above 350 nm. The effect of UV radiation below 350 nm was determined by comparing results under the filter attenuating UV below 350 nm against the control treatment. These contrasts cannot distinguish whether combining different regions of the spectrum had additive or synergistic effects. When multiple tests were used, *p* values were corrected using Holm's correction. A factor, or an interaction between factors, was considered significant in the model when *p* < 0.05. Given that measurements were not taken every day, model predictions, using the function ‘predict. gam’, were used to estimate the mean day of year that phenological events occurred in the different treatments.

## 3 RESULTS

### 3.1 Attenuating blue light delayed leaf out in *Acer platanoides*, but not in herbaceous species

**FIGURE 2:**
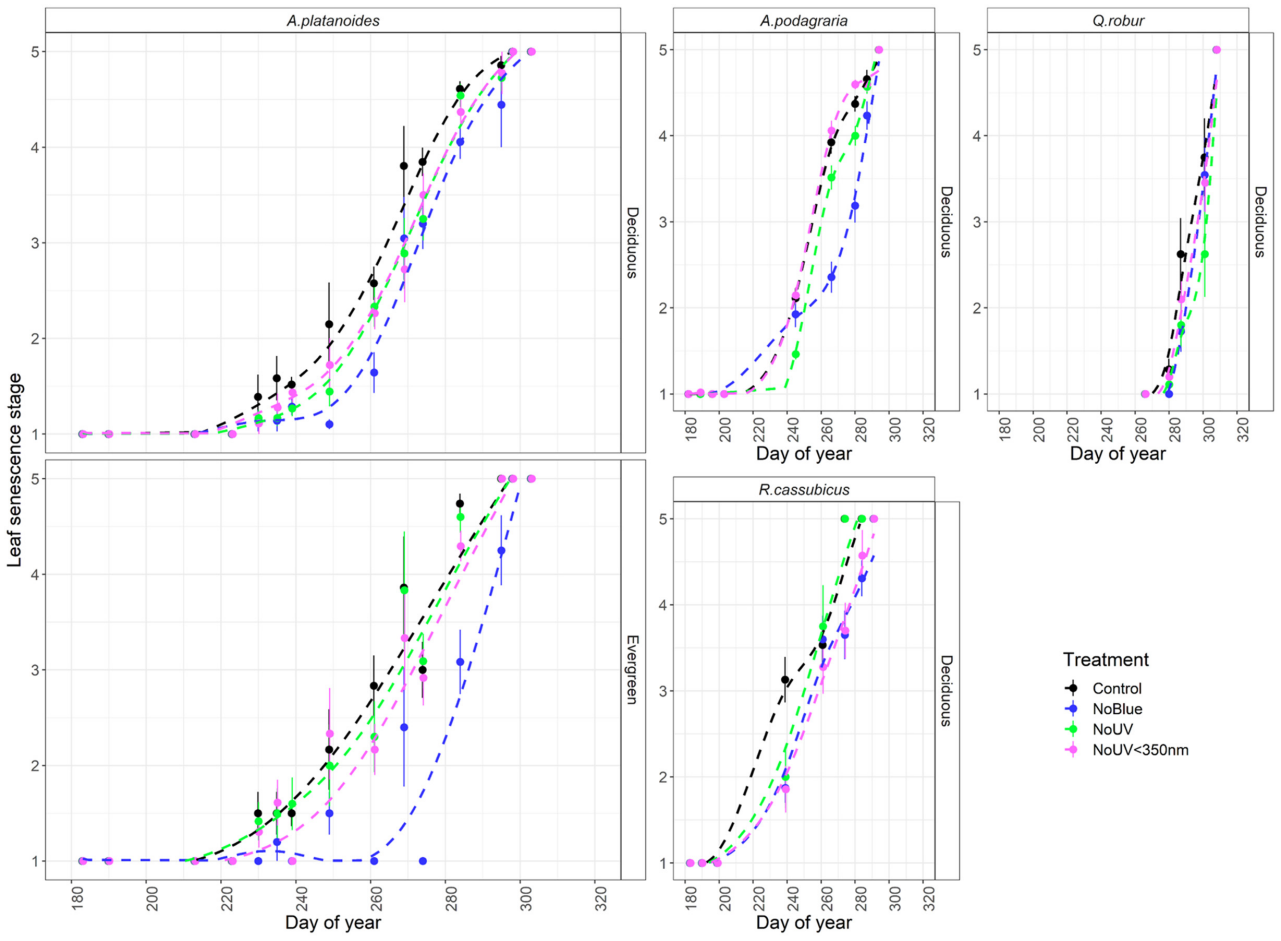
Spring leaf out of plant species growing beneath filters attenuating different regions of the solar spectrum in a forest understorey. For *A. platanoides*, leaf out was scored on a scale of 1–7, whereby 1 = dormant, 2 = bud swelling, 3 = bud split, 4 = leaf tip protruding, 5 = leaf mostly out, 6 = leaf out but not fully expanded, and 7 = fully expanded leaves. For the other plant species, leaf out was scored on a scale of 1–2, whereby 1 = shoot visible, 2 = expanded leaf. Statistical analyses given in Table S3. Dashed lines are fitted using GAM as a smoother in the R package ggplot2 (Wickham, 2016).

There was a significant overall effect of filter treatment on leaf out of *A. platanoides* seedlings (Table S3A,B). Attenuating blue light significantly delayed leaf out, but attenuating UV radiation had no significant effect (Figure 2). The date of bud burst was delayed by 1.4 days when blue light was attenuated, and final leaf out was delayed by 2.8 days when blue light was attenuated. Stand type also had a significant effect on phenology, as bud burst of *A. platanoides* was 5 days later in the evergreen stand than in the deciduous stands (Figure 2). Filter treatments had no significant effect on the leaf out of any herbaceous species (Table S3C–F), although stand type had a significant effect on *A. nemorosa* phenology, whereby its emergence (stage 1) occurred seven days later in the evergreen stand compared to the deciduous stand (Figure 2; Table S3C,D).

### 3.2 Attenuating UV and blue light caused a species‐specific delay in leaf senescence

**FIGURE 3:**
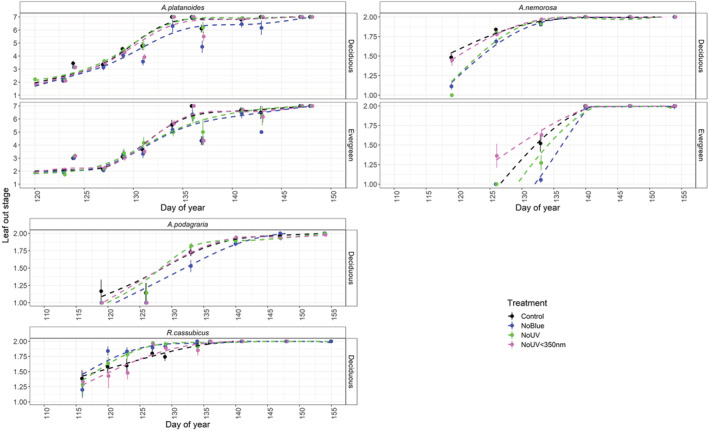
Leaf senescence stage for four understorey plant species growing in deciduous and evergreen stands. Leaf senescence was scored on a scale of 1–5, whereby, 1 = green leaf, 2 = starting to yellow, 3 = mostly yellow, 4 = mostly brown, 5 = leaf fall. Means ± SE presented on the graph, with the individual plot as the unit of replication. Dashed lines are fitted using GAM as a smoother in the R package ggplot2 (Wickham, 2016).

Overall, attenuation of UV radiation below 350‐nm wavelength significantly delayed leaf senescence in *A. platanoides* seedlings (Table S4A,B, Figure 3). The onset of leaf senescence (stage 2) was delayed by 4.1 days when UV radiation below 350 nm was attenuated, although final leaf fall (senescence stage 5) occurred on the same day irrespective of filter treatment (Figure 3). In comparison, attenuating blue light and UV radiation above 350 nm had no overall effect on leaf senescence in *A. platanoides* seedlings (Table S4B). However, in the evergreen stand, attenuating blue light delayed the onset of leaf senescence in *A. platanoides* seedlings by 14.3 days, and final leaf senescence by 7.6 days. Attenuation of blue light also delayed the onset of leaf senescence in herbaceous species *R. cassubicus* by 7.4 days, and final leaf senescence by 7.0 days (Table S4C,D). There was no significant effect of UV radiation below or above 350 nm on leaf senescence in *A. podagraria* (Table S4E,F). This was because, although *A. podagraria* growing under the filter treatment attenuating blue light and UV senesced significantly later than under the control treatment, the effect of the blue light was not significantly different from the no UV treatment (Table S4E,F). This suggests that blue light and UV radiation may have an additive effect on leaf senescence in *A. podagraria*. The onset of leaf senescence in *A. podagraria* was delayed by 5.6 days beneath the filter attenuating blue light and UV radiation compared to the control treatment; however, final leaf senescence occurred on the same day. Our filter treatments had no effect on leaf senescence in *Q. robur* seedlings (Table S4G, Figure 3).

### 3.3 Attenuating blue light reduced flavonol accumulation the most in relatively light‐demanding species

**FIGURE 4:**
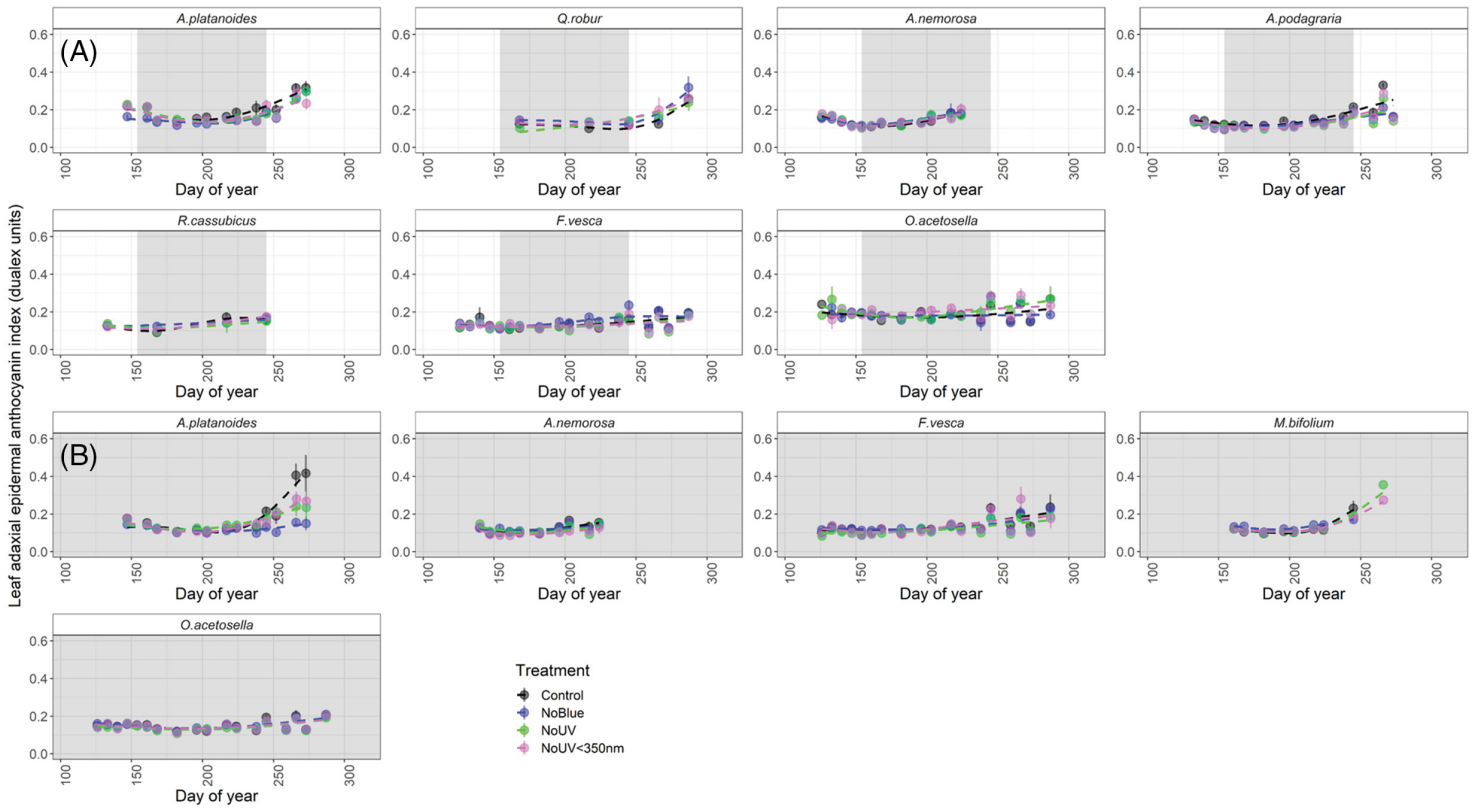
Index of adaxial epidermal flavonol content of eight different plant species in our understorey filter treatments. The grey shaded area represents the period that canopy was closed during the growing season. Means ± SE are presented on the graph, with the individual filter as the unit of replication. Dashed lines fitted using GAM as a smoother in the R package ggplot2 (Wickham, 2016). A, deciduous stands; B, evergreen stands

For most of those plant species we monitored in the understorey of deciduous stands, the general trend was for a high adaxial epidermal flavonol index during spring, followed by a decrease during the period of canopy closure, before flavonols increased again (*Q. robur*, *A. nemorosa*, *A. podagraria*, and *F. vesca*, Figure 4A). Despite the absence of canopy closure and opening, understorey species growing in the evergreen stand displayed a similar but less‐pronounced seasonal pattern in adaxial epidermal flavonols (Figure 4B). Likewise, while our different filter treatments reduced the flavonol index to differing extents, the overall seasonal trends remained consistent among all filter treatments.

Comparing the effects of spectral regions calculated from the differences between pairs of filter treatments, attenuation of blue light generally had the largest effect on adaxial epidermal flavonol accumulation (Table 2, Figure 4A,B). Attenuating blue light significantly reduced adaxial flavonols to a greater extent than any other spectral region in the more light‐demanding species, *A. nemorosa*, *A. platanoides*, *A. podagraria* and *R. cassubicus* (Table S5A–P, Figure 4A,B). Attenuating blue light also reduced adaxial flavonol accumulation in *M. bifolium* and *O. acetosella* (Table 2, Table S5G–L), although its effect was smaller in these shade‐tolerant and wintergreen species (Table 2, Figure 4B).

**TABLE 2:** Break‐down of mean differences in leaf epidermal pigments between filter treatments

Species	Effect	Flavonol diff.	Flavonol%	Sig.	Anthocyanin diff.	Anthocyanin (%)	Sig.
*A. nemorosa*	Blue	−0.25	−30	***	−0.001	−1	ns
	UV > 350	−0.05	−6	**	0.002	+2	ns
	UV < 350	−0.10	−10	ns	−0.001	−1	ns
*A. platanoides*	Blue	−0.24	−35	***	−0.025	−14	*
	UV > 350	0.08	+ 14	***	0.006	+ 4	ns
	UV < 350	−0.17	−22	***	−0.017	−9	***
*A. podagraria*	Blue	−0.28	−38	***	−0.001	−0.1	ns
	UV > 350	0.02	+ 2	ns	−0.006	−5	ns
	UV < 350	−0.13	−15	ns	−0.015	−10	**
*F. vesca*	Blue	−0.03	−5	ns	0.013	+ 10	ns
	UV > 350	−0.06	−9	ns	−0.008	−6	ns
	UV < 350	−0.07	−11	**	−0.005	−4	ns
*M. bifolium*	Blue	−0.02	−7	*	0.009	+ 7	**
	UV > 350	−0.01	−0.1	ns	−0.001	−1	ns
	UV < 350	−0.03	−11	**	0.003	+ 3	ns
*O. acetosella*	Blue	−0.05	−11	***	0.002	+ 1	**
	UV > 350	−0.04	−8	ns	−0.005	−3	ns
	UV < 350	−0.09	−16	ns	0.001	+ 1	**
*Q. robur*	blue	−0.05	−11	ns	0.024	+ 14	ns
	UV > 350	−0.03	−6	ns	−0.020	−10	ns
	UV < 350	−0.25	−36	***	0.042	+ 27	ns
*R. cassubicus*	Blue	−0.21	−35	*	0.006	+ 4	ns
	UV > 350	−0.03	−4	ns	−0.001	−0.1	ns
	UV < 350	−0.08	−12	ns	−0.006	−4	ns

*Note*: Details of the statistical analyses are shown in Tables S5 and S6.

* *p* < 0.05;

** *p* < 0.01;

*** *p* < 0.001.

Attenuating UV radiation above 350 nm significantly reduced the adaxial epidermal flavonol index in *A nemorosa*, and surprisingly increased flavonol accumulation in *A. platanoides*, although the magnitude of both of these effects was small (Table 2, Table S5A–P). Attenuating UV radiation below 350 nm reduced adaxial epidermal flavonols significantly in *A. platanoides*, *F. vesca*, *M. bifolium* and *Q. robur* (Table 2, Table S5A–P), and to a greater extent than UV radiation above 350 nm in these species (Table 2). In *Q. robur* seedlings, the effect of UV radiation below 350 nm on adaxial epidermal flavonol accumulation was even greater than the effect of blue light (Table 2).

Adaxial epidermal flavonol accumulation was generally lower in the evergreen stands than in the deciduous stands, and the absolute (as opposed to relative) effects of our filter treatments were also smaller in the evergreen stands (Table S5A–P, Figure 4A,B). Specifically, the adaxial epidermal flavonol index was significantly lower in the evergreen stands for all those species found beneath both stand types (i.e. *A. nemorosa*, *A. platanoides*, *F. vesca* and *O. acetosella*, Table S5A–P, Figure 4A,B).

### 3.4 Anthocyanins were less responsive than flavonols to seasonal changes in light quality

**FIGURE 5:**
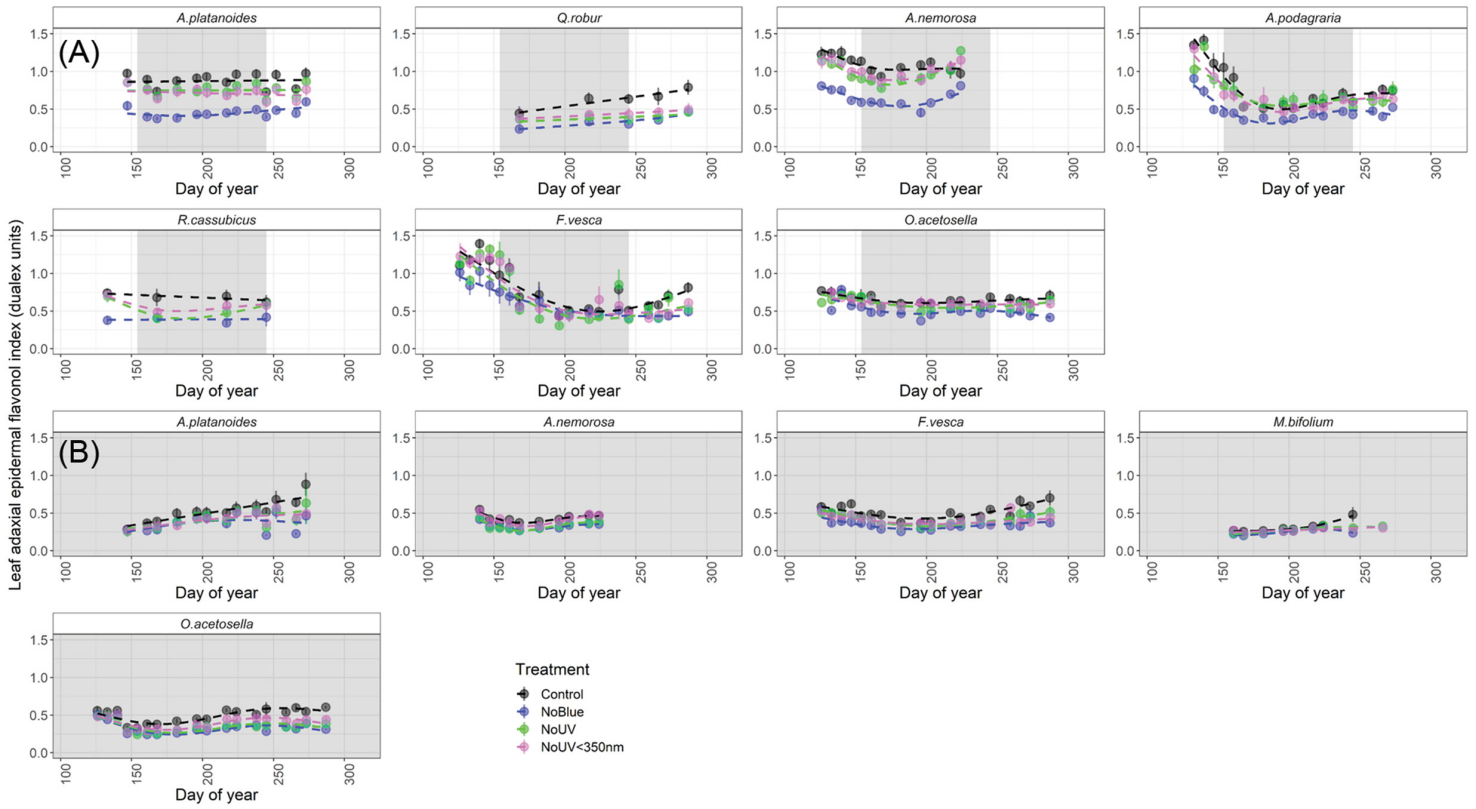
Index of adaxial epidermal anthocyanin content of eight different plant species in our understorey filter treatments in deciduous stands. The grey shaded area represents the period during which the canopy was closed. Means ± SE are presented on the graph, with the individual filter as the unit of replication. Dashed lines are fitted using GAM as a smoother in the R package ggplot2 (Wickham, 2016). A, deciduous stands; B, evergreen stands

The increase in the adaxial epidermal anthocyanin index during understorey leaf senescence was similar to the seasonal trend in epidermal flavonols that we report. Although, unlike flavonols, the adaxial epidermal anthocyanin index was not generally high during early spring. In particular, the largest increases in anthocyanin accumulation during leaf senescence occurred in plants whose leaves senesced latest in the year: *A. platanoides*, *A. podagraria* and *Q. robur* (Weeks 35–40, Figure 5A,B). Overall, seasonal changes in anthocyanins in the adaxial epidermis of leaves were less responsive to our filter treatments than flavonols (Table 2, Figures 4 and 5). Attenuating blue light significantly reduced anthocyanins in *A. platanoides* (Table 2, Table S6A–D). This reduction in the anthocyanin index in *A. platanoides* due to attenuating blue light was greatest during the final weeks of autumn (17.7% reduction compared to the control during Week 38, averaged across stand types, including a 45.5% reduction in the evergreen stand during Week 38, Figure 5B). However, attenuating blue light had the opposite effect of increasing adaxial epidermal anthocyanins accumulation in *M. bifolium* and *O. acetosella* (Table S6I,J,M,N), though these effects were small (Table 2).

The effects on adaxial epidermal anthocyanins of attenuating UV radiation below and above 350 nm were smaller than those of attenuating blue light (Table 2, Table S6A–N, Figures 4 and 5). Attenuating UV radiation below 350 nm reduced the anthocyanin index in *A. platanoides* and *A. podagraria* (Table 2, Table S6A–D, Figure 5A,B), although only to a small degree (Table 2, Figure 5A,B). Attenuating UV radiation above 350 nm had no significant effect on anthocyanin content (Table S6A–N).

## 4 DISCUSSION

### 4.1 Light quality has a small effect on the spring phenology of tree seedlings in the understorey

Blue light advanced bud burst and leaf out by an average of 1.4 and 2.5 days, respectively, in *A. platanoides* seedlings, when grown over two years beneath selective filters in the forest understorey (Figure 2). Attenuating blue light from solar radiation in this sort of filter experiment (Siipola et al., 2015; Wang et al., 2020) also causes a reduction in PAR. However, this small effect of blue light we report is consistent with findings from an experiment using branches of *B. pendula*, *Alnus glutinosa* and *Q. robur* under controlled conditions where PAR was equalised across filter treatments (Brelsford & Robson, 2018). Our findings are also consistent with a recent meta‐analysis, which found UV radiation to have a negligible effect on bud burst (Brelsford, Nybaken, et al., 2019b). Echoing this, a long‐term UV‐B enhancement treatment had no effect on the spring phenology of four dwarf‐shrub species (Phoenix et al., 2001).

We found no strong evidence that light quality affects the emergence and leaf out of the herbaceous species in our study. Possibly because they are submerged below ground during winter and respond to timing of snowmelt and increases in soil temperature, rather than light quality (Price & Waser, 1998; Rice et al., 2018). However, we monitored herbaceous species less often and on a less detailed scale than *A. platanoides*, meaning that subtle differences in their phenology could be overlooked in the present study. As such, increased sampling intensity may be required to further test the effects of light quality on herbaceous species in future studies. Considering our results in context of previous studies, effects of light quality on the spring phenology of plants are most likely small in comparison to other cues and factors such as temperature, chilling and photoperiod (Brelsford, Nybaken, et al., 2019b).

### 4.2 Light quality affects autumn leaf senescence for most of the species measured

The autumn leaf senescence of *A. platanoides*, *A. podagraria* and *R. cassubicus* was delayed by attenuating blue light. Blue light has been shown to enhance photosynthesis beyond simply its contribution to PAR (Goins et al., 1997; Hogewoning et al., 2010; Košvancová‐Zitová et al., 2009; Matsuda et al., 2004; Sæbø et al., 1995). Attenuation of blue light throughout the growing season could reduce photosynthesis, meaning that prolonged leaf retention is required to compensate for reduced carbon gain over the growing season (Chabot & Hicks, 1982; Zhang et al., 2013). Attenuating blue light from solar radiation also reduces PAR, by an average of 34.4% in the no blue no UV filter compared to the control filter in our case. This reduction in PAR could be driving the delayed leaf senescence in response to attenuation of blue light that we report (Lee et al., 2003). This could also explain why attenuating blue light delayed leaf senescence the most in *A. platanoides* seedlings growing in the evergreen stand, where PAR was also lowest (Table S4, Figure 3). Nevertheless, there is some evidence from research into other plant species, such as *Glycine max*, that the blue‐light‐detecting photoreceptors, CRYs, fulfil a specific role in the acceleration of leaf senescence (Meng et al., 2013). Moreover, both CRYs and phytochrome B detect changes in blue light to mediate photoperiod responses in plants via the circadian clock (Fantini & Facella, 2020; Legris et al., 2017; Olsen, 2010). Given that plants growing at higher latitudes may be more responsive to changes in photoperiod (Way & Montgomery, 2015), blue light may also affect plant phenology through CRY‐mediated photoperiodic responses.

In contrast to *A. platanoides*, there was no effect of our filter treatments on the leaf senescence of *Q. robur* seedlings. Autumn leaf senescence in *Q. robur* occurred later and much more abruptly than that of the other species monitored. It appeared to coincide with a sudden drop in temperatures towards the end of autumn 2018 (Week 40, Figure 3, Figure S1). This is consistent with the peak of leaf chlorophyll content found during autumn canopy opening in *Q. robur* (Figure S11). The photosynthetic capacity of *Q. robur* trees is slow to develop through the season (Morecroft et al., 2003) and could indicate that *Q. robur* has a different strategy involving extended carbon assimilation further into autumn compared to other species in our study.

Chlorophyll content was generally higher throughout the growing season and during autumn in *A. platanoides* and *A. podagraria* when blue light was attenuated, compared to the control treatment (Figure S9A,B). For *Q. robur* it was highest in the control treatment (Figure S9A,B). These trends support the leaf senescence observations that were made. However, for *R. cassubicus* this trend was not visible (Figure S9A), demonstrating that visual signs of autumn leaf senescence are the result of multiple pigments, including anthocyanins, and not only the result of chlorophyll degradation.

As well as the effect of blue light, attenuating UV radiation below 350 nm also delayed the onset of leaf senescence in *A. platanoides* (4.1 days delay for UV radiation below 350 nm, and 14.3 days delay for blue light). This agrees with our hypothesis that attenuating UV radiation would delay leaf senescence. This is the first study to find that attenuating ambient solar UV radiation can delay autumn leaf senescence in understorey tree seedlings. However, past research has found that supplemental UV‐B radiation can accelerate leaf senescence in saplings of *Fagus sylvatica* growing in open‐topped chambers compared with those receiving simulated ambient clear‐sky solar UV‐B radiation (Zeuthen et al., 1997). The delay in leaf senescence of *A. platanoides* under attenuated UV radiation below 350 nm could be attributable to leaves suffering less UV‐induced photodamage throughout the growing season under this filter (Zeuthen et al., 1997). However, if this was the mechanism responsible, it is unclear why only leaf senescence in *A. platanoides*, of the species we studied, responded in this way. Interestingly, a long‐term UV‐B enhancement had no effect on the leaf senescence of *Vaccinium myrtillus* and *Vaccinium uliginosum* (Phoenix et al., 2001), suggesting more research is required to unearth the reason for differing species responses.

### 4.3 Flavonol response of shade‐tolerant herbaceous species was less sensitive to changes in light quality than that of more light‐demanding species

We found that more light‐demanding understorey species tend to have the highest epidermal flavonol accumulation throughout the season. These species were also more responsive to our filter treatments than most of the species with overwintering leaves (*O. acetosella* and *M. bifolium*; Figure 4B). This confirms our hypothesis that shade‐tolerant species would be less responsive to the filter treatments. In a study of tropical alpine plants, no significant difference in UV‐screening was found between herbaceous and woody species (Barnes et al., 2017). In contrast to this, a few comparative studies of plant types have suggested that the accumulation of UV‐B absorbing compounds is slightly more responsive to solar radiation in herbaceous species than in woody species, and that evergreen species are the least responsive (Brzezinska & Kozlowska, 2008;Li et al., 2010; Semerdjieva et al., 2003). These differences in flavonoid induction may be offset by higher constitutive contents of UV‐B absorbing compounds in evergreen and woody species compared to herbaceous species (Li et al., 2010; Semerdjieva et al., 2003), possibly related to their greater leaf longevity, which makes investment in flavonoids worthwhile (Semerdjieva et al., 2003). However, we found that despite having shorter leaf longevity, the more light‐demanding plants in our study had a higher flavonol index throughout the season.

Higher baseline investment in flavonols by more light‐demanding species may supplement other rapid photoprotection mechanisms allowing species from this functional group to exploit transient periods of high irradiance and canopy gaps without severe photoinhibition (Takahashi & Badger, 2011). The comparably small response of flavonol index to blue light among shade‐tolerant species may reflect a strategy to maximise light capture under low irradiances (Power et al., 2019). By taking repeated measurements of epidermal flavonoid content on the same leaves, we were unable to harvest leaves to compare temporal patterns against leaf dry matter content in this present study. Repeating this experiment by measuring flavonoid content per unit of leaf dry matter may reveal important differing responses to light quality.

### 4.4 Attenuating blue light cause the largest reduction in adaxial epidermal flavonols for most species

As we hypothesised, out of all our filter treatments, attenuation of blue light caused the largest reduction in adaxial epidermal flavonols in more light‐demanding herbaceous species and deciduous tree seedlings (Figure 4A,B). Interestingly, some previous studies have reported a high correlation between seasonal UV‐A irradiance and flavonol content (Hartikainen et al., 2020) or found no effect of attenuating blue light on flavonols of understorey species (Wang et al., 2020). However, our results are consistent with Siipola et al. (2015), who previously reported a larger effect of blue light than UV radiation on flavonol content in the pea, *Pisum sativum cv. Meteor* grown in full sunlight. Likewise, in a growth room, simulated understorey blue light increased leaf adaxial epidermal flavonol content via CRY photoreceptors under controlled PAR conditions (Brelsford, Morales, et al., 2019a). Accordingly, much of the reduction in flavonols during canopy closure in summer (Figure 4A), could be attributable to the reduction in blue light reaching the understorey. Epidermal flavonols responded differently to our treatments in *Q. robur* compared with most other species. Attenuating UV radiation below 350 nm had the largest effect on *Q. robur* flavonols. This might suggest that at the core of its distribution range, at lower latitudes where UV‐B irradiance is typically higher, UV‐perception is more important as a cue (Bornman et al., 2019).

The seasonal fluctuations in epidermal flavonols in the understorey of the evergreen stands in our study, and the same stands in 2015–2016 (Hartikainen et al., 2020), were much smaller than those in the deciduous stands. Here, we found that the effects of attenuating blue light and UV radiation were also much smaller in the evergreen stands compared to the deciduous. While the lower flavonol content in plants growing in the evergreen understorey can largely be attributed to lower solar irradiance, and its blue and UV regions, the temperature differences between the stands are also likely to contribute to this effect (Davis et al., 2019; Pescheck & Bilger, 2019). Evergreen stands are warmer during the winter and cooler during the summer, because of the insulating effects of a dense closed canopy all year round (Davis et al., 2019). We should also note that ventilation gaps meant that our filter treatments did not totally exclude the attenuated regions of radiation, and the small percentages of solar blue and UV radiation reaching the plants could have had an effect.

### 4.5 Light quality affects leaf flavonol and anthocyanin accumulation in autumn

Until recently, leaf flavonol contents were generally considered not to increase during autumn, because of the presumed inefficiency of investing in leaves at the end of their life (Wilkinson et al., 2002). However, Mattila et al. (2018) found that flavonols increase prior to and during autumn senescence in the tree species *B. pendula* and *Sorbus aucuparia*. Accordingly, we report an autumn increase in the adaxial epidermal flavonol index in *A. platanoides* and *Q. robur* seedlings, as well as understorey *A. podagraria*, and wintergreen *F. vesca* (Weeks 35–40). This increase in flavonols following canopy opening was much smaller, or absent, in plants under filters attenuating UV radiation and blue light (Weeks 35–40, Figure 4A). In autumn, the adaxial epidermal anthocyanin index, like that of epidermal flavonols, tended to increase. However, anthocyanins generally responded less strongly than flavonols to attenuation of blue light and UV radiation.

It is possible that the increase in flavonols and anthocyanins during autumn is due to the increase in solar radiation reaching the understorey after canopy opening (Richardson & O'Keefe, 2009), including shortwave radiation in the blue and UV regions (Hoch et al., 2001). However, there was also an autumnal increase in flavonols and anthocyanins for species growing in the evergreen stand, in the absence of canopy opening (Constabel & Lieffers, 1996; Ross & Flanagan, 1986). This suggests that other factors such as colder temperatures during autumn also contribute to the autumn increase in flavonol and anthocyanin content (Pescheck & Bilger, 2019; Renner & Zohner, 2019). Equally, our results also validate the hypothesis that the increase in anthocyanins may be a part of the leaf senescence timetable and support their role as antioxidants to remove ROS produced during chlorophyll breakdown (Archetti et al., 2009).

## 5 CONCLUSIONS

Both blue light and UV radiation affected the phenology and leaf pigmentation of understorey plants. In general, epidermal flavonols were most responsive to changes in blue light, which makes up a larger portion of the spectrum than UV radiation even in canopy shade. Bud burst was only advanced by blue light in *A. platanoides* seedlings, whose leaf senescence was advanced by UV radiation in the understorey. In contrast, blue light delayed autumn leaf senescence in most of the understorey species including *A. platanoides*. An extended period of canopy shading into autumn, and thus reduced blue light and UV radiation in the understorey, is predicted under climate warming (Buitenwerf et al., 2015; Piao et al., 2019; Vitasse et al., 2009). Considered together with our results, this implies that the growing season in the understorey would be extended for most plants that senesce in autumn. As such, future research should investigate microclimatic interactions between temperature and light quality in understorey plant species.

## AUTHOR CONTRIBUTIONS

Craig C. Brelsford conceived of the experiment and designed the methodology under the supervision of T. Matthew Robson. Tom Paris, Marieke Trasser and Craig C. Brelsford. collected the data. The paper was written, and data analysed, by Craig C. Brelsford. All authors gave editorial input and final approval for publication.

## Supporting information


**Appendix S1** Supporting Information

**Appendix S2** Supporting Information

## Data Availability

Upon publication, data will be available in 4TU.ResearchData at the DOI: 10.4121/19682652.

## References

[ppl13723-bib-0001] Agati, G. & Tattini, M. (2010) Multiple functional roles of flavonoids in photoprotection. New Phytologist, 186, 786–793. 20569414 10.1111/j.1469-8137.2010.03269.x

[ppl13723-bib-0002] Agati, G. , Brunetti, C. , Fini, A. , Gori, A. , Guidi, L. , Landi, M. et al. (2020) Are flavonoids effective antioxidants in plants? Twenty years of our investigation. Antioxidants, 9, 1098. 10.3390/antiox9111098 PMC7695271 33182252

[ppl13723-bib-0003] Aphalo, P.J. , Albert, A. , McLeod, A. , Heikkilä, A. , Gómez, I. , Figueroa, F.L. et al. (2012) Manipulating UV radiation. In: Aphalo, P.J. , Björn, L.O. , McLeod, A. , Robson, T.M. & Rosenqvist, E. (Eds.) Beyond the visible: a handbook of best practice in plant UV photobiology, COST FA0906 UV4growth. Helsinki: University of Helsinki, pp. 35–68.

[ppl13723-bib-0004] Archetti, M. , Döring, T.F. , Hagen, S.B. , Hughes, N.M. , Leather, S.R. , Lee, D.W. et al. (2009) Unravelling the evolution of autumn colours: an interdisciplinary approach. Trends in Ecology and Evolution, 24, 166–173. 19178979 10.1016/j.tree.2008.10.006

[ppl13723-bib-0005] Augspurger, C.K. & Salk, C.F. (2017) Constraints of cold and shade on the phenology of spring ephemeral herb species. Journal of Ecology, 105, 246–254.

[ppl13723-bib-0006] Augspurger, C.K. , Cheeseman, J.M. & Salk, C.F. (2005) Light gains and physiological capacity of understorey woody plants during phenological avoidance of canopy shade. Functional Ecology, 19, 537–546.

[ppl13723-bib-0007] Ballaré, C.L. & Pierik, R. (2017) The shade‐avoidance syndrome: multiple signals and ecological consequences. Plant, Cell and Environment, 40, 2530–2543. 10.1111/pce.12914 28102548

[ppl13723-bib-0008] Ballaré, C.L. , Sánchez, R.A. , Scopel, A.L. , Casal, J.J. & Ghersa, C.M. (1987) Early detection of neighbour plants by phytochrome perception of spectral changes in reflected sunlight. Plant, Cell and Environment, 10, 551–557.

[ppl13723-bib-0009] Barnes, P.W. , Ryel, R.J. & Flint, S.D. (2017) UV screening in native and non‐native plant species in the tropical alpine: implications for climate change‐driven migration of species to higher elevations. Frontiers in Plant Science, 8, 1451. 28878792 10.3389/fpls.2017.01451 PMC5572244

[ppl13723-bib-0010] Bornman, J.F. , Barnes, P.W. , Robson, T.M. , Robinson, S.A. , Jansen, M.A.K. , Ballaré, C.L. et al. (2019) Linkages between stratospheric ozone, UV radiation and climate change and their implications for terrestrial ecosystems. UNEP EEAP report. Photochemical & Photobiological Sciences, 18, 681–716. 30810560 10.1039/c8pp90061b

[ppl13723-bib-0011] Brelsford, C.C. & Robson, T.M. (2018) Blue light advances bud burst in branches of three deciduous tree species under short‐day conditions. Trees, 32, 1157–1164.

[ppl13723-bib-0012] Brelsford, C.C. , Morales, L.O. , Nezval, J. , Kotilainen, T.K. , Hartikainen, S.M. , Aphalo, P.J. et al. (2019a) Do UV‐A radiation and blue light during growth prime leaves to cope with acute high light in photoreceptor mutants of *Arabidopsis thaliana*? Physiologia Plantarum, 165, 537–554. 29704249 10.1111/ppl.12749

[ppl13723-bib-0013] Brelsford, C.C. , Nybaken, L. , Kotilainen, T.K. & Robson, T.M. (2019b) The influence of spectral composition on spring and autumn phenology in trees. Tree Physiology, 39, 925–950. 30901060 10.1093/treephys/tpz026

[ppl13723-bib-0014] Brzezinska, E. & Kozlowska, M. (2008) Effect of sunlight on phenolic compounds accumulation in coniferous plants. Dendrobiology, 59, 3–7.

[ppl13723-bib-0015] Buitenwerf, R. , Rose, L. & Higgins, S.I. (2015) Three decades of multi‐dimensional change in global leaf phenology. Nature Climate Change, 5, 364–368.

[ppl13723-bib-0016] Casal, J.J. (2013) Canopy light signals and crop yield in sickness and in health. ISRN Agronomy, 2013, 1–16.

[ppl13723-bib-0017] Cerovic, Z.G. , Masdoumier, G. , Ghozlen, N.B. & Latouche, G. (2012) A new optical leaf‐clip meter for simultaneous non‐destructive assessment of leaf chlorophyll and epidermal flavonoids. Physiologia Plantarum, 146, 251–260. 22568678 10.1111/j.1399-3054.2012.01639.x PMC3666089

[ppl13723-bib-0018] Chabot, B.F. & Hicks, D.J. (1982) The ecology of leaf life spans. Annual Review of Ecology and Systematics, 13, 229–259.

[ppl13723-bib-0019] Chazdon, R.L. & Pearcy, R.W. (1991) The importance of sunflecks for forest understorey plants. BioScience, 41, 760–766.

[ppl13723-bib-0085] Chuine, I. , & Régnière, J . (2017) Process‐based models of phenology for plants and animals. Annual Review of Ecology, Evolution, and Systematics, 48, 159–182.

[ppl13723-bib-0020] Constabel, A.J. & Lieffers, V.J. (1996) Seasonal patterns of light transmission through boreal mixed wood canopies. Canadian Journal of Forest Research, 26, 1008–1014.

[ppl13723-bib-0021] Davis, K.T. , Dobrowski, S.Z. , Holden, Z.A. , Higuera, P.E. & Abatzoglou, J.T. (2019) Microclimatic buffering in forests of the future: the role of local water balance. Ecography, 42, 1–11.

[ppl13723-bib-0022] Day, T.A. (1993) Relating UV‐B radiation screening effectiveness of foliage to absorbing‐compound concentration and anatomical characteristics in a diverse group of plants. Oecologia, 95, 542–550. 28313295 10.1007/BF00317439

[ppl13723-bib-0023] Day, T.A. , Vogelmann, T.C. & DeLucia, E.H. (1992) Are some plant life forms more effective than others in screening out ultraviolet‐B radiation? Oecologia, 92, 513–519. 28313222 10.1007/BF00317843

[ppl13723-bib-0024] Fantini, E. & Facella, P. (2020) Cryptochromes in the field: how blue light influences crop development. Physiologia Plantarum, 169, 336–346. 32175597 10.1111/ppl.13088

[ppl13723-bib-0025] Federer, C.A. & Tanner, C.B. (1966) Spectral distribution of light in the forest. Ecology, 47, 555–560.

[ppl13723-bib-0026] Feild, T.S. , Lee, D.W. & Holbrook, N.M. (2001) Why leaves turn red in autumn. The role of anthocyanins in senescing leaves of red‐osier dogwood. Plant Physiology, 127, 566–574. 11598230 PMC125091

[ppl13723-bib-0027] Flint, S.D. & Caldwell, M.M. (1998) Solar UV‐B and visible radiation in tropical forest gaps: measurements partitioning direct and diffuse radiation. Global Change Biology, 4, 863–870.

[ppl13723-bib-0086] Flynn, D. F. B. , & Wolkovich, E. M . (2018) Temperature and photoperiod drive spring phenology across all species in a temperate forest community. New Phytologist, 219, 1353‐1362. 29870050 10.1111/nph.15232

[ppl13723-bib-0028] Frelich, L.E. , Machado, J.L. & Reich, P.B. (2003) Fine‐scale environmental variation and structure of understorey plant communities in two old‐growth pine forests. Journal of Ecology, 91, 283–293.

[ppl13723-bib-0029] Goins, G.D. , Yorio, N.C. , Sanwo, M.M. & Brown, C.S. (1997) Photomorphogenesis, photosynthesis, and seed yield of wheat plants grown under red light‐emitting diodes (LEDs) with and without supplemental blue lighting. Journal of Experimental Botany, 48, 1407–1413. 11541074 10.1093/jxb/48.7.1407

[ppl13723-bib-0030] Grant, R.H. , Apostol, K. & Gao, W. (2005) Biologically effective UV‐B exposures of an oak‐hickory forest understorey during leaf‐out. Agricultural and Forest Meteorology, 132, 28–43.

[ppl13723-bib-0031] Grubb, P.J. (1977) The maintenance of species‐richness in plant communities: the importance of the regeneration niche. Biological Reviews, 52, 107–145.

[ppl13723-bib-0032] Hartikainen, S.M. , Jach, A. , Grané, A. & Robson, T.M. (2018) Assessing scale‐wise similarity of curves with a thick pen: as illustrated through comparisons of spectral irradiance. Ecology and Evolution, 8, 10206–10218. 30397459 10.1002/ece3.4496 PMC6206219

[ppl13723-bib-0033] Hartikainen, S.M. , Pieristè, M. , Lassila, J. & Robson, T.M. (2020) Seasonal patterns in spectral irradiance and leaf UV‐A absorbance under forest canopies. Frontiers in Plant Science, 10, 1762. 32133015 10.3389/fpls.2019.01762 PMC7040076

[ppl13723-bib-0034] Heberling, J.M. , Cassidy, S.T. , Fridley, J.D. & Kalisz, S. (2019) Carbon gain phenologies of spring‐flowering perennials in a deciduous forest indicate a novel niche for a widespread invader. New Phytologist, 221, 778–788. 30152089 10.1111/nph.15404

[ppl13723-bib-0035] Hoch, W.A. , Zeldin, E.L. & McCown, B.H. (2001) Physiological significance of anthocyanins during autumnal leaf senescence. Tree Physiology, 21, 1–8. 11260818 10.1093/treephys/21.1.1

[ppl13723-bib-0036] Hoch, W.A. , Singsaas, E.L. & McCown, B.H. (2003) Resorption protection. Anthocyanins facilitate nutrient recovery in autumn by shielding leaves from potentially damaging light levels. Plant Physiology, 133, 1296–1305. 14526111 10.1104/pp.103.027631 PMC281624

[ppl13723-bib-0037] Hogewoning, S.W. , Trouwborst, G. , Maljaars, H. , Poorter, H. , van Ieperen, W. & Harbinson, J. (2010) Blue light dose–responses of leaf photosynthesis, morphology, and chemical composition of *Cucumis sativus* grown under different combinations of red and blue light. Journal of Experimental Botany, 61, 3107–3117. 20504875 10.1093/jxb/erq132 PMC2892149

[ppl13723-bib-0038] Karageorgou, P. & Manetas, Y. (2006) The importance of being red when young: anthocyanins and the protection of young leaves of *Quercus coccifera* from insect herbivory and excess light. Tree Physiology, 26, 613–621. 16452075 10.1093/treephys/26.5.613

[ppl13723-bib-0039] Košvancová‐Zitová, M. , Urban, O. , Navrátil, M. , Špunda, V. , Robson, T.M. & Marek, M.V. (2009) Blue radiation stimulates photosynthetic induction in *Fagus sylvatica* L. Photosynthetica, 47, 388.

[ppl13723-bib-0084] Kudo, G. , Ida, T. Y. , & Tani, T . (2008) Linkages between phenology, pollination, photosynthesis, and reproduction in deciduous forest understory plants. Ecology, 89, 321–331. 18409422 10.1890/06-2131.1

[ppl13723-bib-0087] Landhäusser, S. M. , Stadt, K. J. , & Lieffers, V. J . (1997) Photosynthetic strategies of summergreen and evergreen understory herbs of the boreal mixedwood forest. Oecologia, 112, 173–178. 28307567 10.1007/s004420050297

[ppl13723-bib-0040] Lee, D.W. (2002) Anthocyanins in autumn leaf senescence. Advances in Botanical Research, 37, 147–165.

[ppl13723-bib-0041] Lee, D.W. , O'Keefe, J. , Holbrook, N.M. & Field, T.S. (2003) Pigment dynamics and autumn leaf senescence in a New England deciduous forest, eastern USA. Ecological Research, 18, 677–694.

[ppl13723-bib-0042] Legris, M. , Nieto, C. , Sellaro, R. , Prat, S. & Casal, J.J. (2017) Perception and signalling of light and temperature cues in plants. Plant Journal, 90, 683–697. 10.1111/tpj.13467 28008680

[ppl13723-bib-0043] Leuchner, M. , Fabian, P. & Werner, H. (2005) Spectral multichannel monitoring of radiation within a mature mixed forest. Plant Biology, 7, 619–627. 16388465 10.1055/s-2005-872971

[ppl13723-bib-0044] Li, F.R. , Peng, S.L. , Chen, B.M. & Hou, Y.P. (2010) A meta‐analysis of the responses of woody and herbaceous plants to elevated ultraviolet‐B radiation. Acta Oecologica, 36, 1–9.

[ppl13723-bib-0046] Matsuda, R. , Ohashi‐Kaneko, K. , Fujiwara, K. , Goto, E. & Kurata, K. (2004) Photosynthetic characteristics of rice leaves grown under red light with or without supplemental blue light. Plant and Cell Physiology, 45, 1870–1874. 15653806 10.1093/pcp/pch203

[ppl13723-bib-0047] Mattila, H. , Valev, D. , Havurinne, V. , Khorobrykh, S. , Virtanen, O. , Antinluoma, M. et al. (2018) Degradation of chlorophyll and synthesis of flavonols during autumn senescence—the story told by individual leaves. AoB Plants, 10, ply028. 29977486 10.1093/aobpla/ply028 PMC6007487

[ppl13723-bib-0048] Meng, Y. , Li, H. , Wang, Q. , Liu, B. & Lin, C. (2013) Blue light–dependent interaction between cryptochrome2 and CIB1 regulates transcription and leaf senescence in soybean. The Plant Cell, 25, 4405–4420. 24272488 10.1105/tpc.113.116590 PMC3875726

[ppl13723-bib-0049] Messier, C. , Parent, S. & Bergeron, Y. (1998) Effects of overstory and understorey vegetation on the understorey light environment in mixed boreal forests. Journal of Vegetation Science, 9, 511–520.

[ppl13723-bib-0050] Morecroft, M.D. , Stokes, V.J. & Morison, J.I.L. (2003) Seasonal changes in the photosynthetic capacity of canopy oak (Quercus robur) leaves: the impact of slow development on annual carbon uptake. International Journal of Biometeorology, 47, 221–226. 12733054 10.1007/s00484-003-0173-3

[ppl13723-bib-0051] Niinemets, Ü. (2010) A review of light interception in plant stands from leaf to canopy in different plant functional types and in species with varying shade tolerance. Ecological Research, 25, 693–714.

[ppl13723-bib-0052] Olsen, J.E. (2010) Light and temperature sensing and signaling in induction of bud dormancy in woody plants. Plant molecular biology, 73, 37–47. 20213333 10.1007/s11103-010-9620-9

[ppl13723-bib-0053] Pedersen, E.J. , Miller, D.L. , Simpson, G.L. & Ross, N. (2019) Hierarchical generalized additive models in ecology: an introduction with mgcv. PeerJ, 7, e6876. 31179172 10.7717/peerj.6876 PMC6542350

[ppl13723-bib-0054] Pescheck, F. & Bilger, W. (2019) High impact of seasonal temperature changes on acclimation of photoprotection and radiation‐induced damage in field grown Arabidopsis thaliana. Plant Physiology and Biochemistry, 134, 129–136. 30093294 10.1016/j.plaphy.2018.07.037

[ppl13723-bib-0055] Phoenix, G.K. , Gwynn‐Jones, D. , Callaghan, T.V. , Sleep, D. & Lee, J.A. (2001) Effects of global change on a sub‐Arctic heath: effects of enhanced UV‐B radiation and increased summer precipitation. Journal of Ecology, 89, 256–267.

[ppl13723-bib-0056] Piao, S. , Liu, Q. , Chen, A. , Janssens, I.A. , Fu, Y. , Dai, J. et al. (2019) Plant phenology and global climate change: current progresses and challenges. Global Change Biolology, 25, 1922–1940. 10.1111/gcb.14619 30884039

[ppl13723-bib-0057] Pinheiro, J. , Bates, D. , DebRoy, S. , Sarkar, D. , Heisterkamp, S. , Van Willigen, B. , & Maintainer, R. (2017) Package ‘nlme’. Linear and Nonlinear Mixed Effects Models, version, 3‐1.

[ppl13723-bib-0058] Power, S.C. , Verboom, G.A. , Bond, W.J. & Cramer, M.D. (2019) Does a trade‐off between trait plasticity and resource conservatism contribute to the maintenance of alternate stable states? New Phytologist, 223, 1809–1819. 31177527 10.1111/nph.15981

[ppl13723-bib-0059] Price, M.V. & Waser, N.M. (1998) Effects of experimental warming on plant reproductive phenology in a subalpine meadow. Ecology, 79, 1261–1271.

[ppl13723-bib-0060] Rai, N. , Neugart, S. , Yan, Y. , Wang, F. , Siipola, S.M. , Lindfors, A.V. et al. (2019) How do cryptochromes and UVR8 interact in natural and simulated sunlight? Journal of Experimental Botany, 70, 4975–4990. 31100755 10.1093/jxb/erz236 PMC6760287

[ppl13723-bib-0061] Rai, N. , O'Hara, A. , Farkas, D. , Safronov, O. , Ratanasopa, K. , Wang, F. et al. (2020) The photoreceptor UVR8 mediates the perception of both UV‐B and UV‐A wavelengths up to 350 nm of sunlight with responsivity moderated by cryptochromes. Plant, Cell and Environment, 43, 1513–1527. 10.1111/pce.13752 32167576

[ppl13723-bib-0062] Renner, S.S. & Zohner, C.M. (2019) The occurrence of red and yellow autumn leaves explained by regional differences in insolation and temperature. New Phytologist, 224, 1464–1471. 31070794 10.1111/nph.15900

[ppl13723-bib-0063] Rice, K.E. , Montgomery, R.A. , Stefanski, A. , Rich, R.L. & Reich, P.B. (2018) Experimental warming advances phenology of ground layer plants at the boreal‐temperate forest ecotone. American Journal of Botany, 105, 851–861. 29874393 10.1002/ajb2.1091

[ppl13723-bib-0064] Richardson, A.D. & O'Keefe, J. (2009) Phenological differences between understorey and overstory. In: Phenology of ecosystem processes. New York: Springer, pp. 87–117.

[ppl13723-bib-0088] Ross, M. S. , Flanagan, L. B. , & Roi, G. H. L . (1986) Seasonal and successional changes in light quality and quantity in the understory of boreal forest ecosystems. Canadian Journal of Botany, 64, 2792–2799.

[ppl13723-bib-0065] Saarinen, T. , Rasmus, S. , Lundell, R. , Kauppinen, O.K. & Hänninen, H. (2016) Photosynthetic and phenological responses of dwarf shrubs to the depth and properties of snow. Oikos, 125, 364–373.

[ppl13723-bib-0066] Sæbø, A. , Krekling, T. & Appelgren, M. (1995) Light quality affects photosynthesis and leaf anatomy of birch plantlets in vitro. Plant Cell, Tissue and Organ Culture, 41(177), 185.

[ppl13723-bib-0067] Semerdjieva, S.I. , Sheffield, E. , Phoenix, G.K. , Gwynn‐Jones, D. , Callaghan, T.V. & Johnson, G.N. (2003) Contrasting strategies for UV‐B screening in sub‐Arctic dwarf shrubs. Plant, Cell and Environment, 26, 957–964. 10.1046/j.1365-3040.2003.01029.x 12803622

[ppl13723-bib-0068] Siipola, S.M. , Kotilainen, T. , Sipari, N. , Morales, L.O. , Lindfors, A.V. , Robson, T.M. et al. (2015) Epidermal UV‐A absorbance and whole‐leaf flavonoid composition in pea respond more to solar blue light than to solar UV radiation. Plant, Cell and Environment, 38, 941–952. 10.1111/pce.12403 25040832

[ppl13723-bib-0069] Takahashi, S. & Badger, M.R. (2011) Photoprotection in plants: a new light on photosystem II damage. Trends in Plant Science, 16, 53–60. 21050798 10.1016/j.tplants.2010.10.001

[ppl13723-bib-0070] Teissier du Crois, E. , Le Tacon, F. , Nepveu, G. , Pardé, J. & Timbal, J. (1981) Le hêtre. Paris: Département des Recherches Forestières, INRA, 613 pp.

[ppl13723-bib-0071] Turnbull, T.L. , Barlow, A.M. & Adams, M.A. (2013) Photosynthetic benefits of ultraviolet‐A to *Pimelea ligustrina*, a woody shrub of sub‐alpine Australia. Oecologia, 173, 375–385. 23529370 10.1007/s00442-013-2640-9

[ppl13723-bib-0072] Vezina, P.E. & Boulter, D.W.K. (1966) The spectral composition of near ultraviolet and visible radiation beneath forest canopies. Canadian Journal of Botany, 44, 1267–1284.

[ppl13723-bib-0073] Vitasse, Y. , Porté, A.J. , Kremer, A. , Michalet, R. & Delzon, S. (2009) Responses of canopy duration to temperature changes in four temperate tree species: relative contributions of spring and autumn leaf phenology. Oecologia, 161, 187–198. 19449036 10.1007/s00442-009-1363-4

[ppl13723-bib-0074] Wang, Q.W. , Robson, T.M. , Pieristè, M. , Oguro, M. , Oguchi, R. , Murai, Y. et al. (2020) Testing trait plasticity to the spectral composition of sunlight in forest understorey species differing in shade tolerance. Journal of Ecology, 108, 1923–1940.

[ppl13723-bib-0075] Way, D.A. & Montgomery, R.A. (2015) Photoperiod constraints on tree phenology, performance and migration in a warming world. Plant, Cell & Environment, 38, 1725–1736. 10.1111/pce.12431 25142260

[ppl13723-bib-0076] Wickham, H. (2016) ggplot2: elegant graphics for data analysis. New York: Springer‐Verlag, 224 pp.

[ppl13723-bib-0077] Wilkinson, D.M. , Sherratt, T.N. , Phillip, D.M. , Wratten, S.D. , Dixon, A.F. & Young, A.J. (2002) The adaptive significance of autumn leaf colours. Oikos, 99, 402–407.

[ppl13723-bib-0078] Wolkovich, E.M. & Cleland, E.E. (2011) The phenology of plant invasions: a community ecology perspective. Frontiers in Ecology and the Environment, 9, 287–294.

[ppl13723-bib-0079] Wood, S.N. , & Wood, M.S. (2015) Package ‘mgcv’. R package version, 1‐7.

[ppl13723-bib-0081] Zeuthen, J. , Mikkelsen, T.N. , Paludan‐Müller, G. & Ro‐Poulsen, H. (1997) Effects of increased UV‐B radiation and elevated levels of tropospheric ozone on physiological processes in European beech (*Fagus sylvatica*). Physiologia Plantarum, 100, 281–290.

[ppl13723-bib-0082] Zhang, Y.J. , Yang, Q.Y. , Lee, D.W. , Goldstein, G. & Cao, K.F. (2013) Extended leaf senescence promotes carbon gain and nutrient resorption: importance of maintaining winter photosynthesis in subtropical forests. Oecologia, 173, 721–730. 23636462 10.1007/s00442-013-2672-1

[ppl13723-bib-0083] Zuur, A.F. , Ieno, E.N. & Elphick, C.S. (2010) A protocol for data exploration to avoid common statistical problems. Methods in Ecology and Evolution, 1, 3–14.

