# Supplementary material for: Understorey light quality affects leaf pigments and leaf phenology in different plant functional types
Source: Physiol Plant. 2022 Jun 14;174(3):e13723. doi: 10.1111/ppl.13723 (PMC9328371; doi:10.1111/ppl.13723)
Supplement: Supplementary file 1 — Appendix S1 Supporting Information [file PPL-174-0-s002.docx]

**Supplemental Materials**

**Tables**

**Table S1:** Photon irradiance for different spectral regions and photon ratios at solar noon.

**Table S2:** The Leaf Area Index (LAI) of the replicate plots in each deciduous and evergreen stands

**Figures**

**Fig. S1:** Daily air temperature in an open area at Lammi Biological Station (2017-2018).

**Fig. S2:** Daily precipitation in an open area at Lammi Biological Station (2017-2018).

**Fig. S3:** Daily mean UV Index at Jokioinen Ilmala (2017-2018)

**Fig. S4:** Daily incident PAR from an open area at Lammi Biological Station (2015-2019).

**Fig. S5:** Example of the filter structure in the field.

**Fig. S6:** Soil moisture (% vol) at 0-15-cm depth under different filter treatments and outside the filters in the forest understory of each experimental stand.

**Fig. S7:** Air temperature 10-20 cm height above the ground under different filter treatments and outside the filters in the forest understory of each experimental stand.

**Fig. S8:** Comparison of time-series temperature data for air temperature under the filters in the forest understorey and outside the stands (April to October 2018).

**Fig. S9:** Leaf chlorophyll content (optical index – arbitrary units) of eight understorey plant species measured with a Dualex during 2017 and 2018.

**Fig. S10:** Leaf senescence in autumn for different understorey species under a deciduous canopy in 2017 and 2018.

**Statistical Tables (S3-S6)**

**Table S3:** Analyses of Leaf Out Phenology (Tables S3 A-F)

**Table S4**: Analysis of Leaf Senescence Phenology (Tables S4 A-G)

**Table S5** Analyses of Leaf Epidermal Flavanols (Tables S5 A-P)

**Table S6:** Analyses of Leaf Epidermal Anthocyanins (Tables S6 A-P)

**Table S1**. Photon irradiance for different spectral regions and photon ratios at solar noon. Measurements under clear sky conditions from each of the forest stands across different dates before and after canopy leaf flush. Type refers to the location of the diffuser in the light environment created by a sunfleck, semi-shade of a canopy leaf or full shade. Table is split over three pages.

| **Date** | **Stand** | **Type** | **PAR(PPFD) µmol m^-2^ s^-1^** | **UV-B(q)  µmol m^-2^ s^-1^** | **UV-A(q)  µmol m^-2^ s^-1^** | **Blue(q)  µmol m^-2^ s^-1^** | **Green(q)  µmol m^-2^ s^-1^** | **Red(q)  µmol m^-2^ s^-1^** | **Far-red(q)** | | **UV-B:PAR** | **R:FR** | **B:G** |
| --- | --- | --- | --- | --- | --- | --- | --- | --- | --- | --- | --- | --- | --- |
| 2015/04/25 | *Betula* Old | sun | 882.01 | 0.60 | 61.33 | 171.36 | 214.48 | 202.95 | 169.82 | 0.72 | | 1.03 | 0.80 |
| 2015/04/25 | *Betula* Young | sun | 963.39 | 0.78 | 68.98 | 187.93 | 234.01 | 220.80 | 184.84 | 0.83 | | 1.03 | 0.80 |
| 2015/04/25 | *Betula* Old | sun | 86.42 | 0.06 | 6.53 | 17.11 | 21.20 | 19.42 | 19.08 | 0.84 | | 0.82 | 0.81 |
| 2015/04/25 | *Quercus* | sun | 1095.77 | 0.72 | 74.85 | 212.52 | 266.88 | 251.82 | 209.18 | 0.67 | | 1.04 | 0.80 |
| 2015/05/22 | *Betula* Old | sun | 729.89 | 0.58 | 50.04 | 139.58 | 180.12 | 166.65 | 150.14 | 0.88 | | 0.92 | 0.78 |
| 2015/05/22 | *Betula* Young | sun | 1043.08 | 0.88 | 68.66 | 197.39 | 258.05 | 238.81 | 218.31 | 0.85 | | 0.93 | 0.76 |
| 2015/05/22 | *Picea abies* | sun | 521.44 | 0.34 | 32.76 | 99.37 | 127.40 | 120.81 | 102.11 | 0.66 | | 1.01 | 0.78 |
| 2015/05/22 | *Quercus* | sun | 1049.05 | 0.99 | 76.37 | 205.79 | 256.66 | 237.81 | 197.89 | 0.95 | | 1.03 | 0.80 |
| 2015/07/05 | *Betula* Old | sun | 570.20 | 0.44 | 35.51 | 107.38 | 141.09 | 131.27 | 123.61 | 0.76 | | 0.84 | 0.76 |
| 2015/07/05 | *Betula* Young | sun | 746.76 | 0.47 | 46.37 | 140.97 | 184.46 | 172.30 | 162.10 | 0.63 | | 0.89 | 0.76 |
| 2015/07/05 | *Picea abies* | sun | 871.34 | 0.53 | 52.84 | 165.20 | 213.09 | 202.49 | 166.01 | 0.64 | | 1.05 | 0.78 |
| 2015/07/05 | *Quercus* | sun | 756.19 | 0.46 | 46.81 | 141.70 | 187.45 | 174.26 | 163.35 | 0.61 | | 0.89 | 0.76 |
| 2015/08/21 | *Betula* Old | sun | 286.61 | 0.23 | 19.09 | 54.92 | 71.05 | 65.26 | 65.67 | 0.87 | | 0.82 | 0.77 |
| 2015/08/21 | *Betula* Young | sun | 814.06 | 0.43 | 49.53 | 153.27 | 200.55 | 188.62 | 167.27 | 0.53 | | 0.98 | 0.76 |
| 2015/08/21 | *Quercus* | sun | 561.53 | 0.47 | 35.39 | 105.99 | 138.33 | 129.84 | 118.32 | 0.83 | | 0.95 | 0.77 |
| 2015/04/25 | *Betula* Old | shade | 144.05 | 0.34 | 22.47 | 35.02 | 34.41 | 27.70 | 26.72 | 2.35 | | 0.87 | 1.02 |
| 2015/04/25 | *Betula* Young | shade | 206.41 | 0.42 | 26.10 | 45.90 | 49.49 | 42.93 | 39.96 | 2.11 | | 0.90 | 0.94 |
| 2015/04/25 | *Picea abies* | shade | 20.36 | 0.04 | 2.86 | 4.65 | 5.02 | 4.00 | 6.33 | 2.01 | | 0.50 | 0.93 |
| 2015/04/25 | *Quercus* | shade | 190.61 | 0.32 | 24.79 | 43.78 | 46.02 | 38.47 | 33.77 | 1.68 | | 0.98 | 0.96 |
| 2015/05/22 | *Betula* Old | shade | 85.15 | 0.26 | 14.03 | 19.29 | 22.71 | 14.74 | 29.07 | 3.05 | | 0.38 | 0.85 |
| 2015/05/22 | *Betula* Young | shade | 111.83 | 0.24 | 13.55 | 21.78 | 30.60 | 21.35 | 44.87 | 2.10 | | 0.35 | 0.71 |
| 2015/05/22 | *Picea abies* | shade | 29.90 | 0.04 | 3.43 | 6.44 | 7.41 | 6.15 | 9.52 | 1.49 | | 0.50 | 0.88 |
| 2015/05/22 | *Quercus* | shade | 93.13 | 0.42 | 19.69 | 24.66 | 23.37 | 14.90 | 22.01 | 4.53 | | 0.52 | 1.05 |
| 2015/07/05 | *Betula* Old | shade | 49.07 | 0.17 | 7.79 | 10.74 | 13.87 | 8.24 | 28.23 | 3.43 | | 0.20 | 0.77 |
| 2015/07/05 | *Betula* Young | shade | 40.21 | 0.10 | 6.00 | 8.19 | 11.85 | 6.73 | 29.69 | 2.66 | | 0.15 | 0.69 |
| 2015/07/05 | *Picea abies* | shade | 17.61 | 0.03 | 2.68 | 4.06 | 4.44 | 3.32 | 6.46 | 3.15 | | 0.32 | 0.98 |
| 2015/07/05 | *Quercus* | shade | 51.45 | 0.13 | 7.32 | 9.41 | 15.47 | 8.64 | 31.05 | 2.44 | | 0.19 | 0.61 |

**Table S1**. (continued)

| **Date** | **Stand** | **Type** | **PAR(PPFD) µmol m^-2^ s^-1^** | **UV-B(q)  µmol m^-2^ s^-1^** | **UV-A(q)  µmol m^-2^ s^-1^** | **Blue(q)  µmol m^-2^ s^-1^** | **Green(q)  µmol m^-2^ s^-1^** | **Red(q)  µmol m^-2^ s^-1^** | **Far-red(q)** | | **UV-B:PAR** | **R:FR** | **B:G** |
| --- | --- | --- | --- | --- | --- | --- | --- | --- | --- | --- | --- | --- | --- |
| 2015/08/21 | *Betula* Old | shade | 17.30 | 0.06 | 3.10 | 3.98 | 5.11 | 2.59 | 15.54 | 3.61 | | 0.12 | 0.77 |
| 2015/08/21 | *Betula* Young | shade | 38.74 | 0.08 | 5.01 | 8.34 | 10.52 | 7.11 | 24.29 | 1.97 | | 0.21 | 0.79 |
| 2015/08/21 | *Quercus* | shade | 19.69 | 0.07 | 3.30 | 4.25 | 6.01 | 2.95 | 18.86 | 3.78 | | 0.11 | 0.71 |
| 2015/05/22 | *Betula* Old | leaf | 391.82 | 0.43 | 31.41 | 76.52 | 97.53 | 87.01 | 86.05 | 1.12 | | 0.84 | 0.79 |
| 2015/05/22 | *Betula* Young | leaf | 557.49 | 0.55 | 40.17 | 105.89 | 139.36 | 125.46 | 127.66 | 1.01 | | 0.80 | 0.76 |
| 2015/05/22 | *Picea abies* | leaf | 324.60 | 0.16 | 20.84 | 62.50 | 79.49 | 74.52 | 64.30 | 0.52 | | 0.98 | 0.79 |
| 2015/05/22 | *Betula* Old | leaf | 627.18 | 0.59 | 43.36 | 120.92 | 155.45 | 141.89 | 135.78 | 1.03 | | 0.81 | 0.78 |
| 2015/07/05 | *Betula* Young | leaf | 199.24 | 0.20 | 15.07 | 38.13 | 50.71 | 43.94 | 59.11 | 1.21 | | 0.54 | 0.75 |
| 2015/07/05 | *Picea abies* | leaf | 112.52 | 0.07 | 8.25 | 21.95 | 27.61 | 25.48 | 24.30 | 1.29 | | 0.72 | 0.83 |
| 2015/07/05 | *Quercus* | leaf | 304.50 | 0.26 | 21.78 | 56.90 | 77.11 | 68.18 | 78.30 | 0.95 | | 0.65 | 0.72 |
| 2015/08/21 | *Betula* Old | leaf | 149.75 | 0.19 | 11.61 | 29.17 | 37.39 | 33.42 | 40.38 | 1.51 | | 0.64 | 0.79 |
| 2015/08/21 | *Betula* Young | leaf | 140.86 | 0.03 | 9.33 | 26.67 | 35.79 | 31.43 | 43.50 | 0.24 | | 0.59 | 0.75 |
| 2015/08/21 | *Quercus* | leaf | 207.29 | 0.19 | 14.51 | 39.61 | 51.94 | 46.68 | 53.32 | 0.98 | | 0.65 | 0.75 |
| 2016/04/21 | *Picea abies* | leaf | 29.01 | 0.04 | 3.08 | 6.18 | 7.12 | 6.06 | 7.73 | 2.30 | | 0.50 | 0.95 |
| 2016/04/22 | *Picea abies* | leaf | 820.64 | 0.59 | 56.77 | 158.26 | 198.64 | 188.35 | 160.14 | 0.76 | | 0.95 | 0.80 |
| 2016/05/09 | *Betula* Old | leaf | 292.21 | 0.44 | 26.07 | 59.05 | 72.52 | 63.14 | 60.70 | 1.53 | | 0.85 | 0.82 |
| 2016/05/09 | *Betula* Young | leaf | 263.14 | 0.42 | 22.20 | 50.59 | 66.92 | 56.92 | 66.33 | 1.69 | | 0.65 | 0.76 |
| 2016/05/09 | *Picea abies* | leaf | 17.45 | 0.07 | 3.00 | 4.57 | 4.42 | 2.86 | 5.13 | 3.91 | | 0.40 | 1.04 |
| 2016/05/09 | *Quercus* | leaf | 395.78 | 0.57 | 33.64 | 79.00 | 96.72 | 87.73 | 77.23 | 1.61 | | 0.92 | 0.83 |
| 2016/05/24 | *Betula* Old | leaf | 79.49 | 0.21 | 9.43 | 16.71 | 21.04 | 15.37 | 28.44 | 2.89 | | 0.38 | 0.80 |
| 2016/05/24 | *Picea abies* | leaf | 25.38 | 0.07 | 3.43 | 5.74 | 6.18 | 5.06 | 6.07 | 4.49 | | 0.59 | 0.90 |
| 2016/05/25 | *Betula* Young | leaf | 70.96 | 0.12 | 6.63 | 13.93 | 19.05 | 14.32 | 30.22 | 1.97 | | 0.31 | 0.72 |
| 2016/05/25 | *Quercus* | leaf | 62.81 | 0.14 | 6.43 | 11.90 | 18.26 | 11.52 | 33.37 | 2.22 | | 0.23 | 0.65 |
| 2016/04/21 | *Betula* Old | shade | 99.63 | 0.28 | 18.29 | 26.30 | 23.73 | 17.29 | 18.15 | 2.76 | | 0.74 | 1.11 |
| 2016/04/21 | *Betula* Young | shade | 114.31 | 0.35 | 20.72 | 29.31 | 27.09 | 20.54 | 21.60 | 3.10 | | 0.74 | 1.08 |
| 2016/04/21 | *Picea abies* | shade | 8.86 | 0.03 | 1.96 | 2.40 | 2.21 | 1.37 | 3.83 | 3.80 | | 0.25 | 1.09 |
| 2016/04/21 | *Quercus* | shade | 94.56 | 0.33 | 19.44 | 26.08 | 22.40 | 15.56 | 16.53 | 3.53 | | 0.72 | 1.16 |

**Table S1**. (continued)

| **Date** | **Stand** | **Type** | **PAR(PPFD) µmol m^-2^ s^-1^** | **UV-B(q)  µmol m^-2^ s^-1^** | **UV-A(q)  µmol m^-2^ s^-1^** | **Blue(q)  µmol m^-2^ s^-1^** | **Green(q)  µmol m^-2^ s^-1^** | **Red(q)  µmol m^-2^ s^-1^** | **Far-red(q)** | | **UV-B:PAR** | **R:FR** | **B:G** |
| --- | --- | --- | --- | --- | --- | --- | --- | --- | --- | --- | --- | --- | --- |
| 2016/05/09 | *Betula* Old | shade | 138.91 | 0.37 | 18.85 | 32.72 | 35.60 | 25.22 | 30.22 | 2.64 | | 0.65 | 0.92 |
| 2016/05/09 | *Betula* Young | shade | 112.76 | 0.35 | 15.03 | 24.46 | 30.58 | 20.13 | 37.01 | 3.10 | | 0.39 | 0.80 |
| 2016/05/09 | *Picea abies* | shade | 16.23 | 0.07 | 3.01 | 4.27 | 4.10 | 2.63 | 4.97 | 4.27 | | 0.38 | 1.04 |
| 2016/05/09 | *Quercus* | shade | 132.60 | 0.45 | 21.85 | 34.19 | 33.14 | 22.53 | 24.04 | 3.39 | | 0.73 | 1.03 |
| 2016/05/24 | *Betula* Old | shade | 54.63 | 0.19 | 7.70 | 11.89 | 15.15 | 9.55 | 25.50 | 3.48 | | 0.26 | 0.78 |
| 2016/05/24 | *Picea abies* | shade | 12.72 | 0.05 | 2.08 | 3.14 | 3.31 | 2.13 | 4.93 | 3.78 | | 0.31 | 0.95 |
| 2016/05/25 | *Betula* Young | shade | 37.07 | 0.09 | 4.73 | 7.85 | 10.77 | 6.26 | 23.27 | 2.51 | | 0.18 | 0.73 |
| 2016/05/25 | *Quercus* | shade | 49.93 | 0.13 | 5.82 | 9.73 | 14.99 | 8.47 | 29.96 | 2.61 | | 0.19 | 0.65 |
| 2016/04/21 | *Betula* Old | Sun | 472.38 | 0.38 | 36.80 | 94.03 | 114.23 | 106.23 | 91.95 | 0.95 | | 0.92 | 0.84 |
| 2016/04/21 | *Betula* Young | Sun | 803.60 | 0.62 | 57.14 | 155.88 | 194.48 | 183.62 | 156.96 | 0.78 | | 0.95 | 0.80 |
| 2016/04/21 | *Picea abies* | Sun | 111.58 | 0.06 | 7.33 | 21.39 | 27.20 | 25.55 | 23.81 | 1.22 | | 0.72 | 0.85 |
| 2016/04/21 | *Quercus* | Sun | 849.79 | 0.61 | 58.58 | 164.07 | 205.82 | 194.83 | 165.26 | 0.74 | | 0.95 | 0.80 |
| 2016/04/29 | *Picea abies* | Sun | 602.18 | 0.31 | 31.94 | 109.44 | 146.48 | 142.95 | 119.43 | 0.51 | | 0.99 | 0.75 |
| 2016/05/09 | *Betula* Old | Sun | 664.57 | 0.56 | 41.62 | 121.28 | 162.32 | 156.14 | 136.64 | 0.85 | | 0.96 | 0.75 |
| 2016/05/09 | *Betula* Young | Sun | 848.53 | 0.76 | 51.85 | 154.41 | 209.06 | 197.96 | 179.73 | 0.91 | | 0.90 | 0.74 |
| 2016/05/09 | *Picea abies* | Sun | 485.77 | 0.30 | 25.68 | 86.50 | 117.83 | 116.88 | 96.65 | 0.62 | | 1.01 | 0.73 |
| 2016/05/09 | *Quercus* | Sun | 843.52 | 0.72 | 53.91 | 156.65 | 204.97 | 197.68 | 164.99 | 0.87 | | 1.00 | 0.77 |
| 2016/05/24 | *Betula* Old | Sun | 672.95 | 0.57 | 41.08 | 125.14 | 165.78 | 155.75 | 138.96 | 0.86 | | 0.92 | 0.75 |
| 2016/05/24 | *Picea abies* | Sun | 485.89 | 0.38 | 28.56 | 90.24 | 118.83 | 113.39 | 92.28 | 0.77 | | 1.04 | 0.76 |
| 2016/05/25 | *Betula* Young | Sun | 510.21 | 0.31 | 27.23 | 90.84 | 125.68 | 121.35 | 113.55 | 0.65 | | 0.86 | 0.72 |

**Table S2**. The Leaf Area Index (LAI) of the replicate plots in each deciduous and evergreen stands. Data calculated from hemispherical photographs taken following (Hartikainen et al., 2018) on 09/06/2017.

| **Stand** | **Plot** | **Date** | **LAI (mean)** | **LAI (SE)** |
| --- | --- | --- | --- | --- |
| *Betula pendula* old | 1 | 09/06/2017 | 1.697 | 0.006 |
| *Betula pendula* old | 2 | 09/06/2017 | 1.983 | 0.023 |
| *Betula pendula* old | 3 | 09/06/2017 | 2.063 | 0.051 |
| *Betula pendula* young | 1 | 09/06/2017 | 2.480 | 0.073 |
| *Betula pendula* young | 2 | 09/06/2017 | 2.343 | 0.107 |
| *Betula pendula* young | 3 | 09/06/2017 | 2.273 | 0.081 |
| *Picea abies* | 1 | 09/06/2017 | 3.697 | 0.137 |
| *Picea abies* | 2 | 09/06/2017 | 3.660 | 0.116 |
| *Picea abies* | 3 | 09/06/2017 | 3.833 | 0.122 |
| *Picea abies* | 4 | 09/06/2017 | 3.320 | 0.094 |
| *Picea abies* | 5 | 09/06/2017 | 3.203 | 0.118 |
| *Picea abies* | 6 | 09/06/2017 | 3.407 | 0.124 |
| *Quercus robur* | 1 | 09/06/2017 | 1.500 | 0.006 |
| *Quercus robur* | 2 | 09/06/2017 | 1.530 | 0.006 |
| *Quercus robur* | 3 | 09/06/2017 | 1.453 | 0.047 |

**Supplemental Figures**


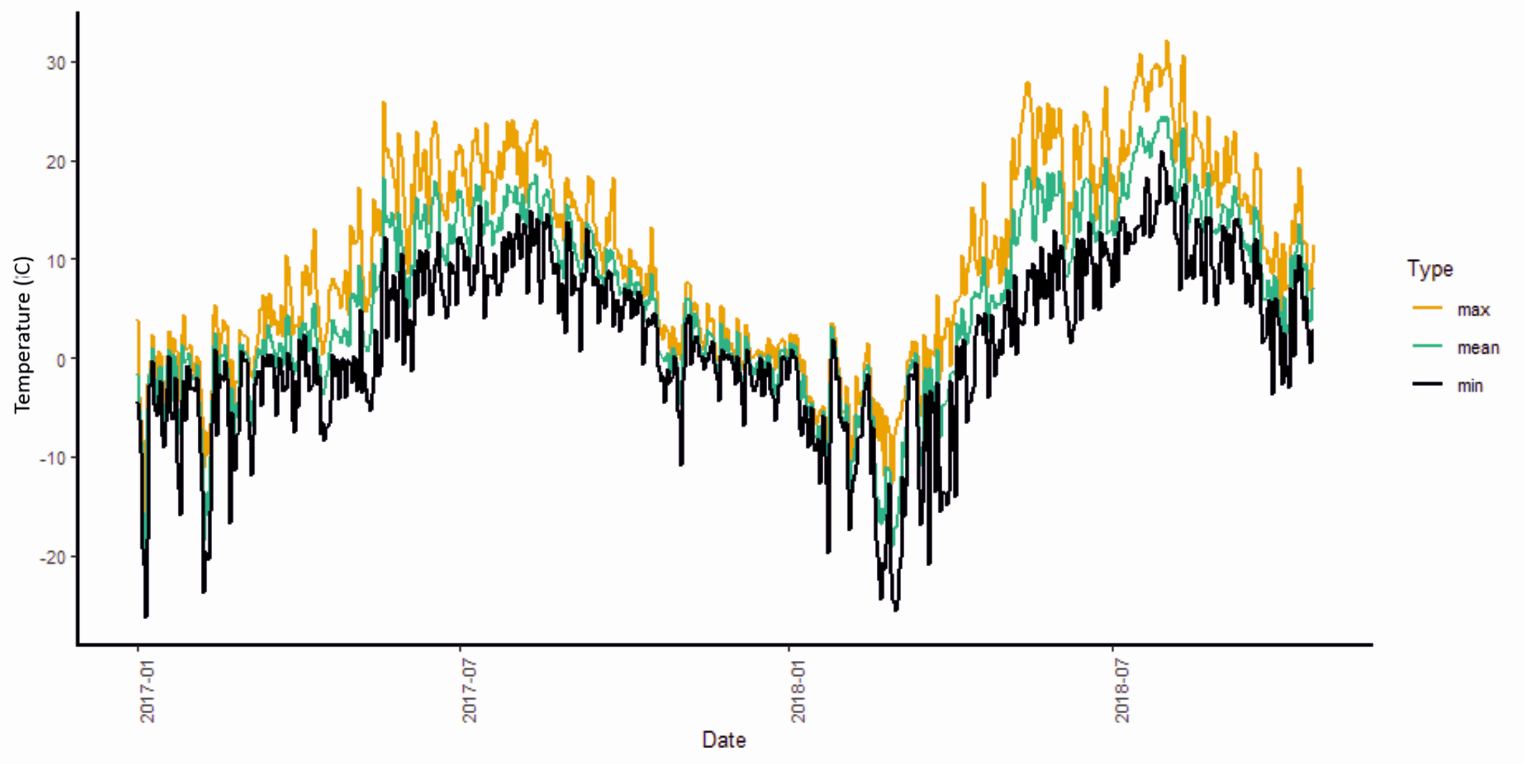


**Fig. S1**. Daily air temperature in an open area at Lammi Biological Station (2017-2018). The data were recorded at the site and processed by the Finnish Meteorological Institute (downloaded from <https://www.ilmatieteenlaitos.fi/havaintojen-lataus#!/>).


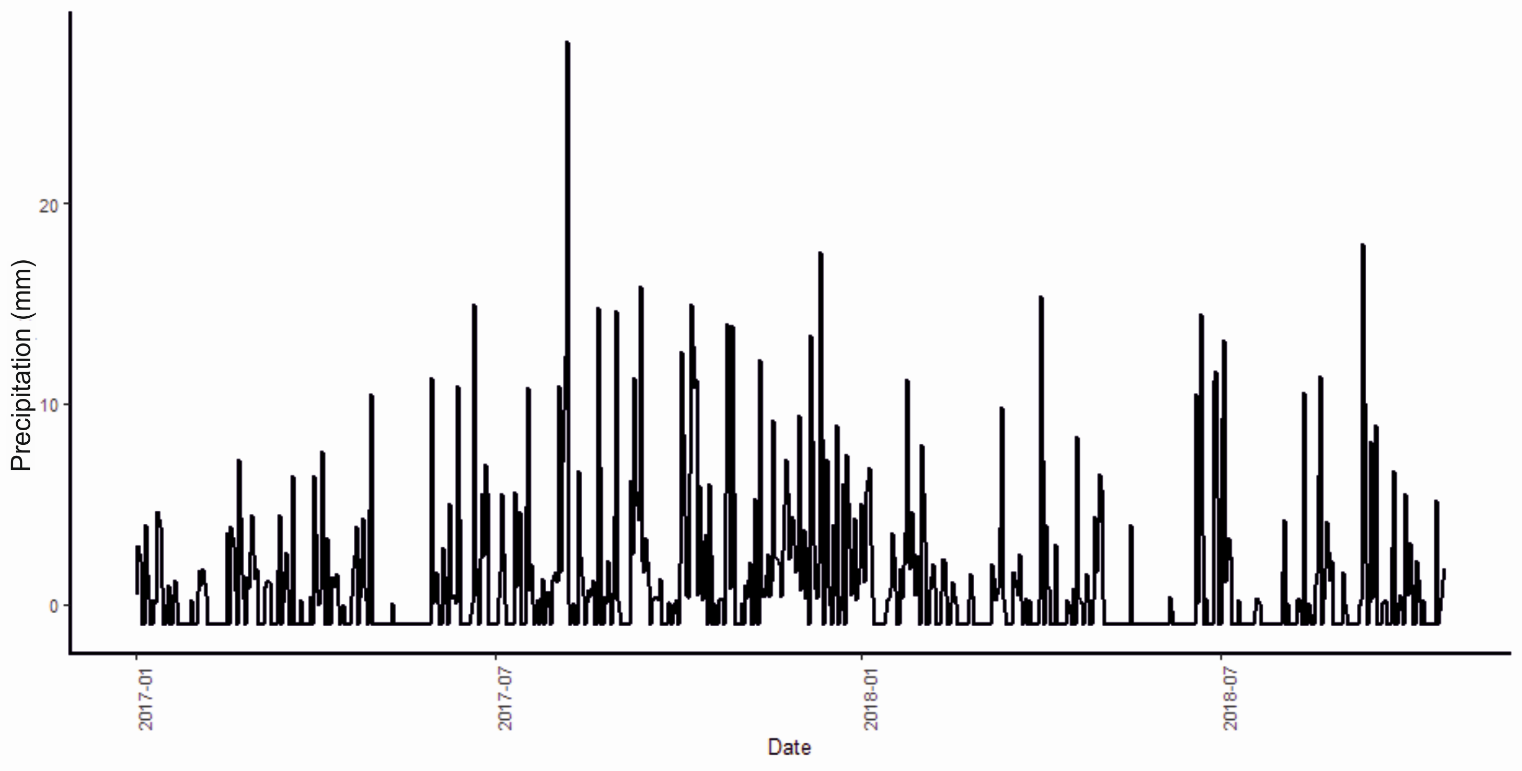


**Fig. S2**. Daily precipitation in an open area at Lammi Biological Station (2017-2018). The data were recorded at the site and processed by the Finnish Meteorological Institute (downloaded from <https://www.ilmatieteenlaitos.fi/havaintojen-lataus#!/>).


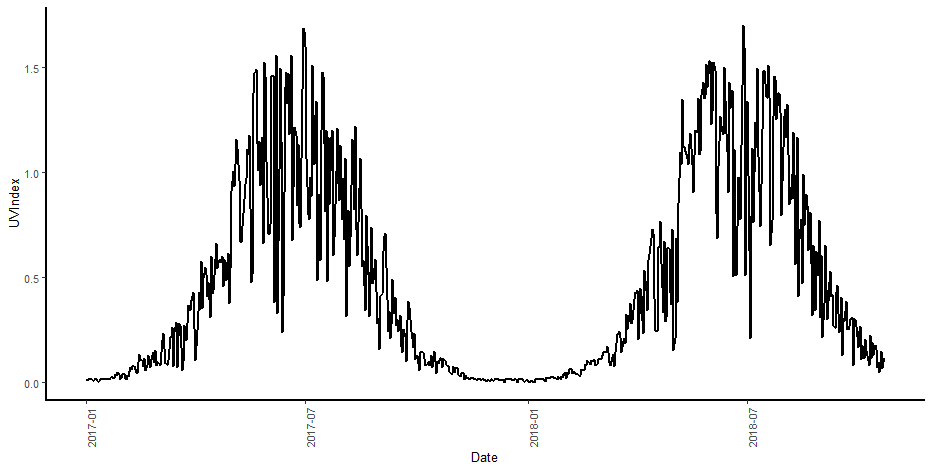


**Fig. S3.** Daily mean UV Index at Jokioinen Ilmala (2017-2018); the closest FMI station to the study site at Lammi where UV irradiance data is recorded. The data were recorded at the site and processed by the Finnish Meteorological Institute (downloaded from <https://www.ilmatieteenlaitos.fi/havaintojen-lataus#!/>).


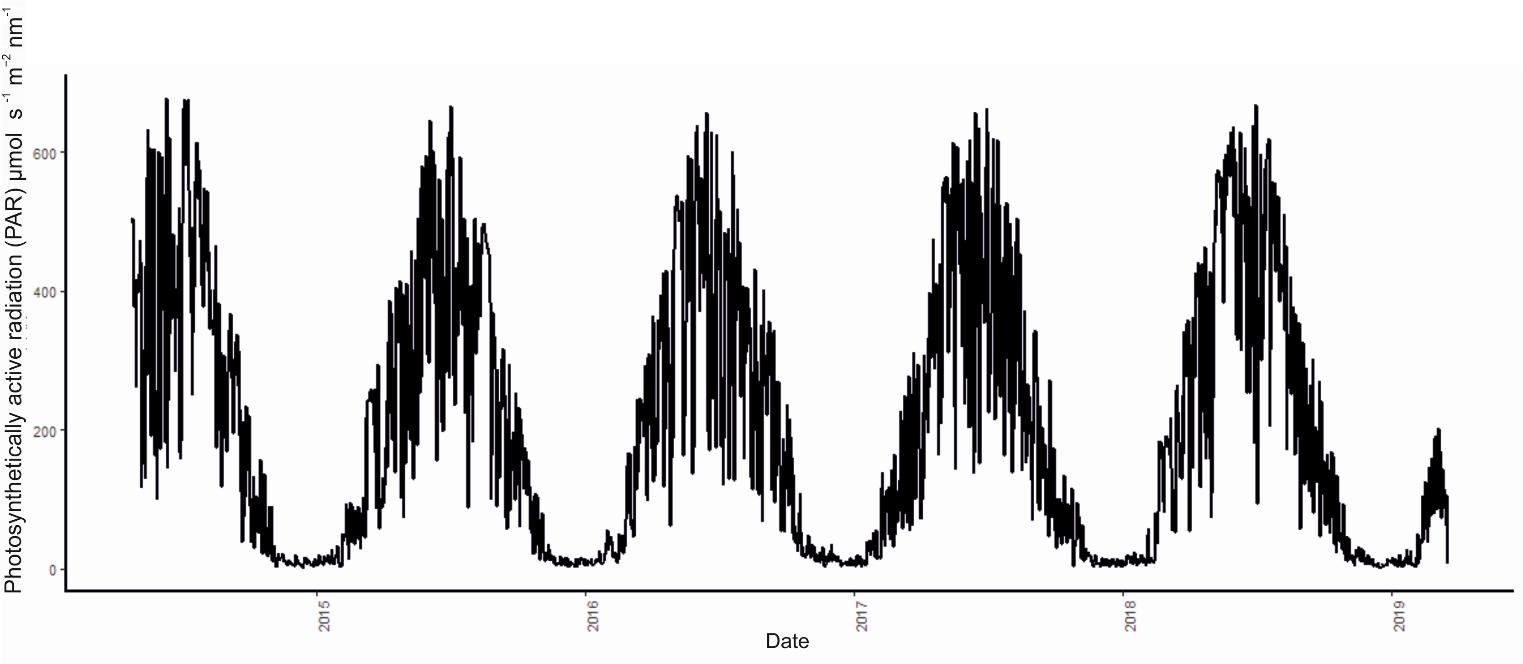


**Fig. S4**. Daily incident PAR from an open area at Lammi Biological Station (2015-2019). Data show values integrated over 10-minute intervals, and illustrate the effects of changing cloudiness on incoming solar radiation. Measurements recorded with a PQS1 PAR Quantum Sensor (Kipp & Zonen, Delft, Netherlands).


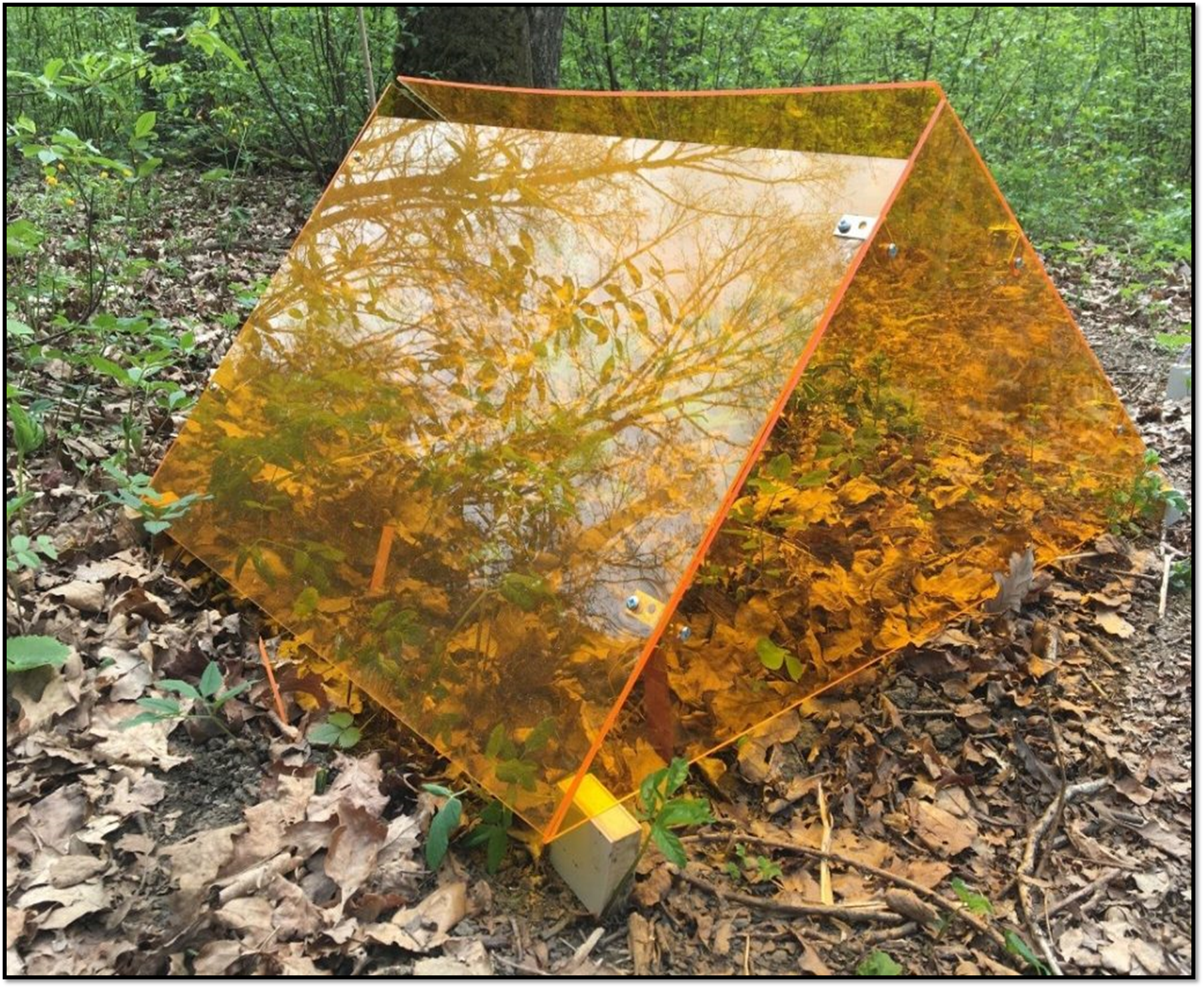


**Fig. S5**. Example of the filter structure in the field. This filter is a “No blue and UV treatment” installed in a deciduous stand with a *Q. robur* canopy. All filter treatments had identical filter structures (88-cm length × 60-cm width × 40-cm in height) with ventilation slots at the apex and around the base.


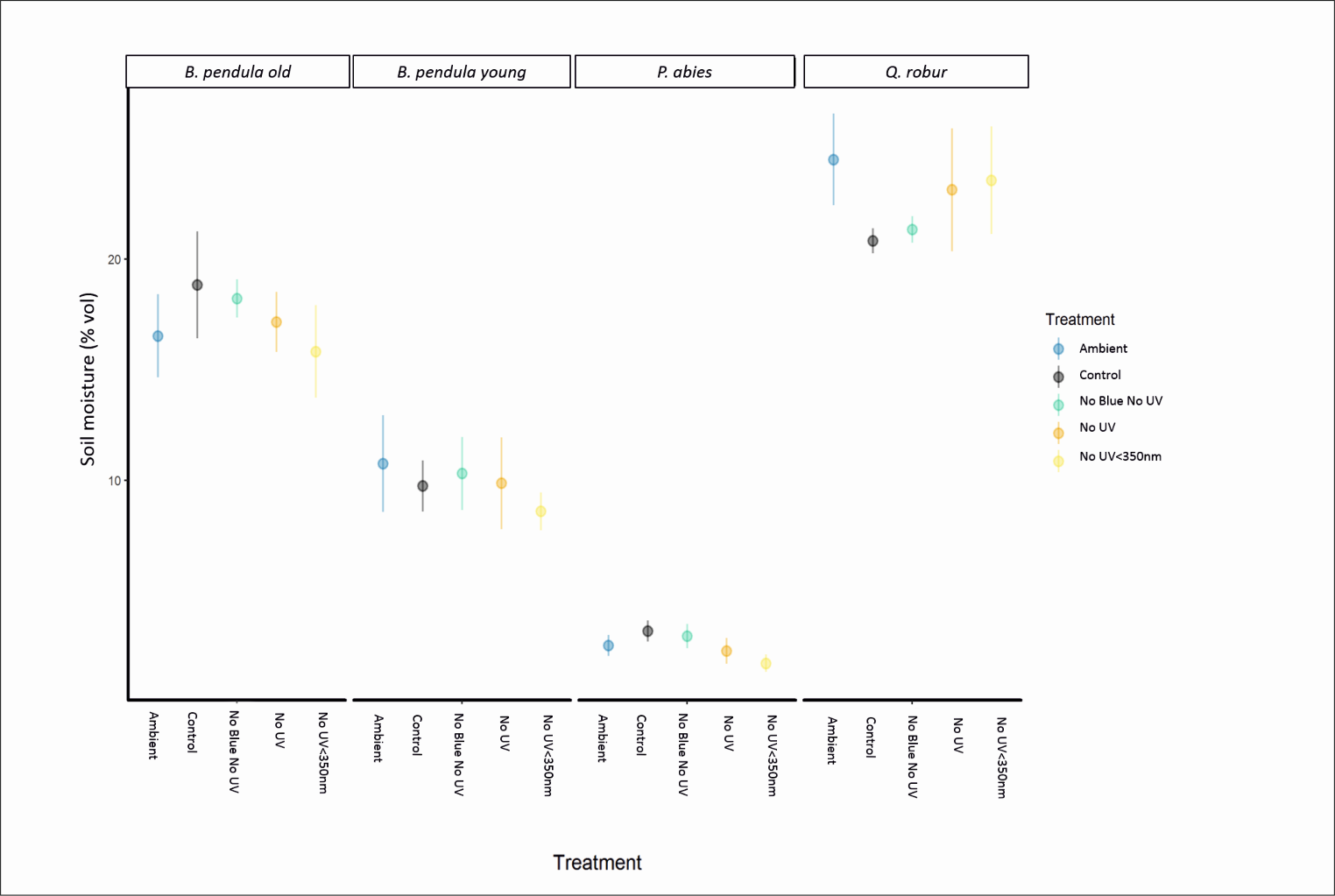


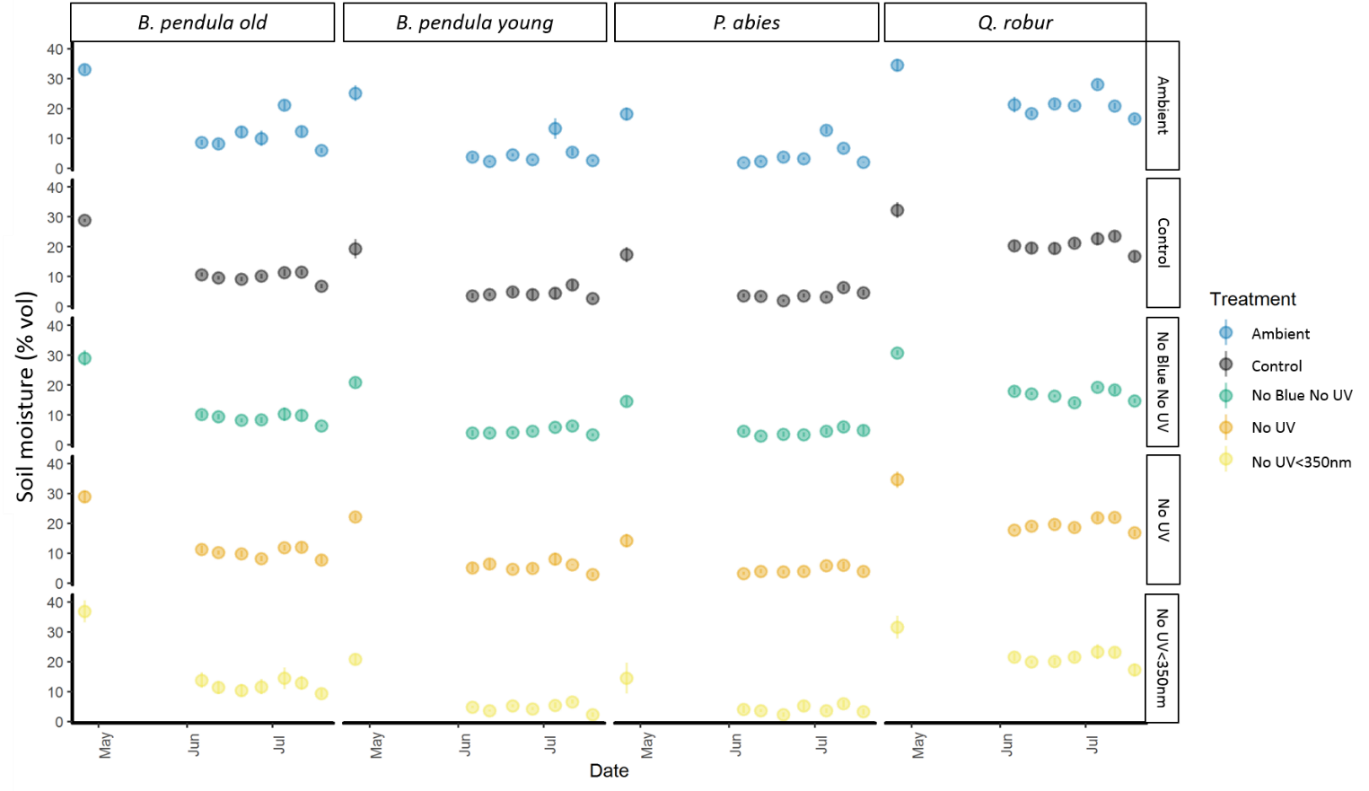


**Fig. S6**. Soil moisture (% vol) at 0-15-cm depth under different filter treatments and outside the filters in the forest understory of each experimental stand. The upper-panel measurement was a measurement from 17^th^ June 2017, whilst the lower panel shows repeated measurements of soil moisture during the spring and summer of 2018.


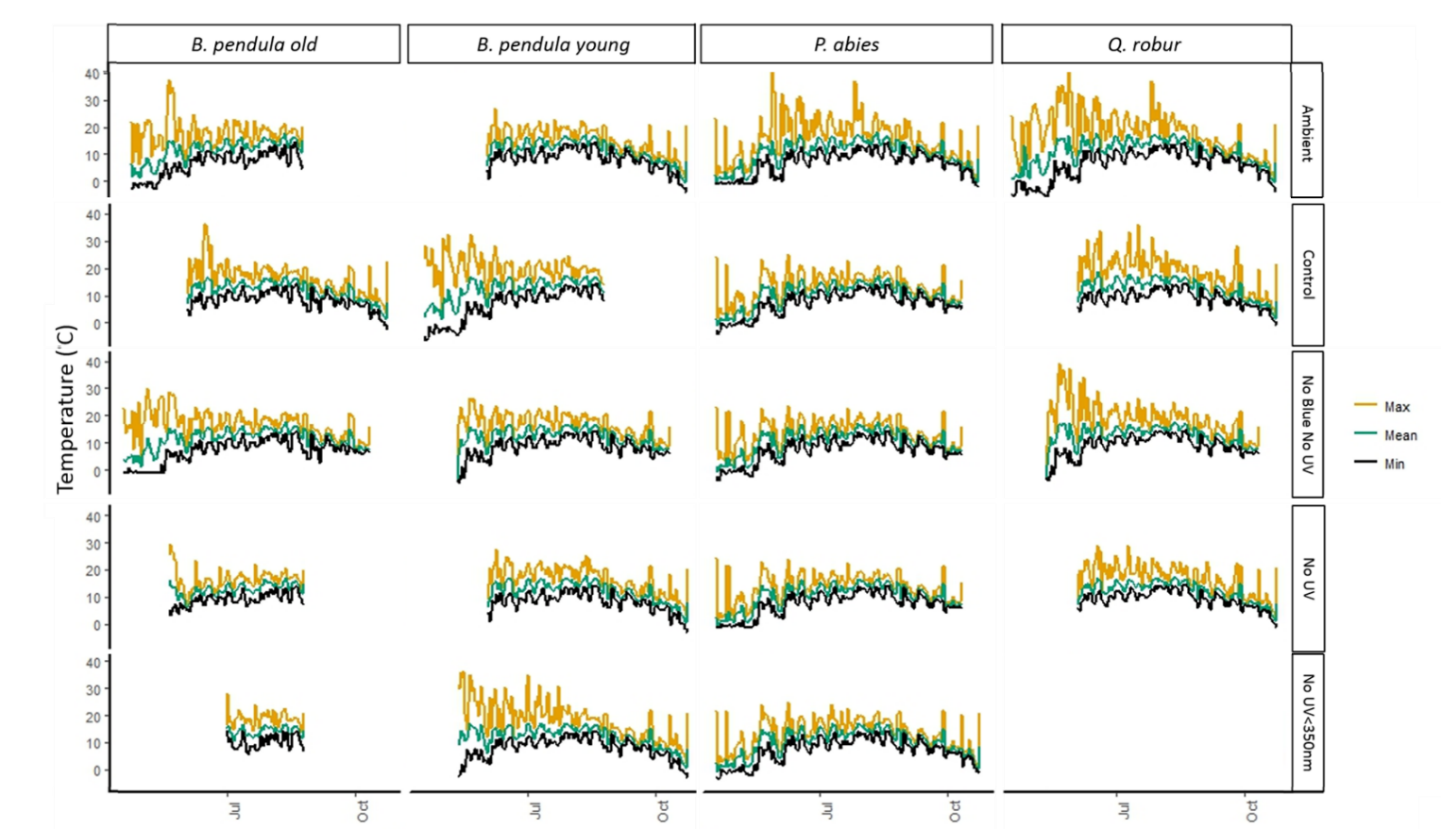


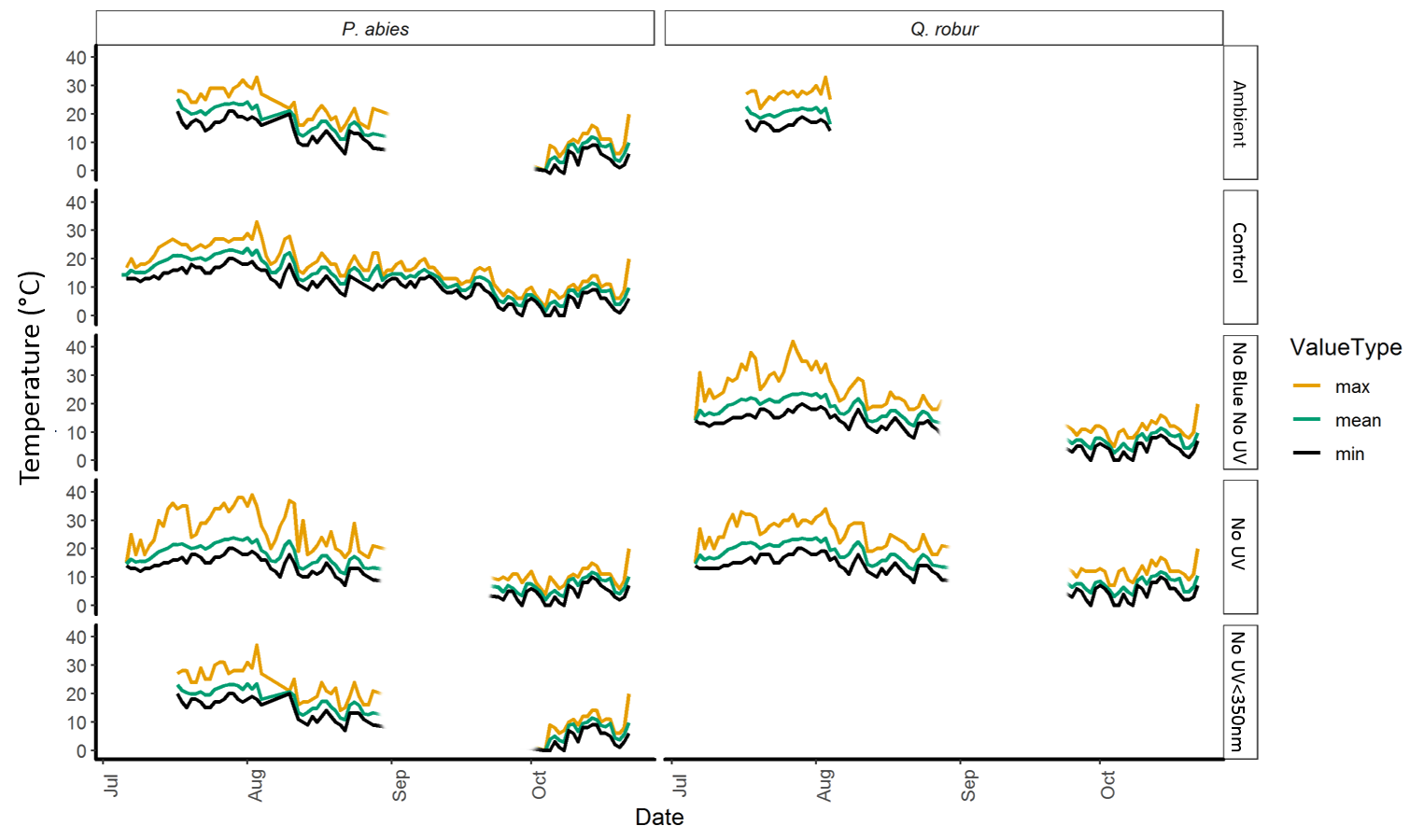
**Fig. S7**. Air temperature 10-20 cm height above the ground under different filter treatments and outside the filters in the forest understory of each experimental stand. The upper panel shows repeated measurements during the spring and summer of 2017 and the lower panel 2018.


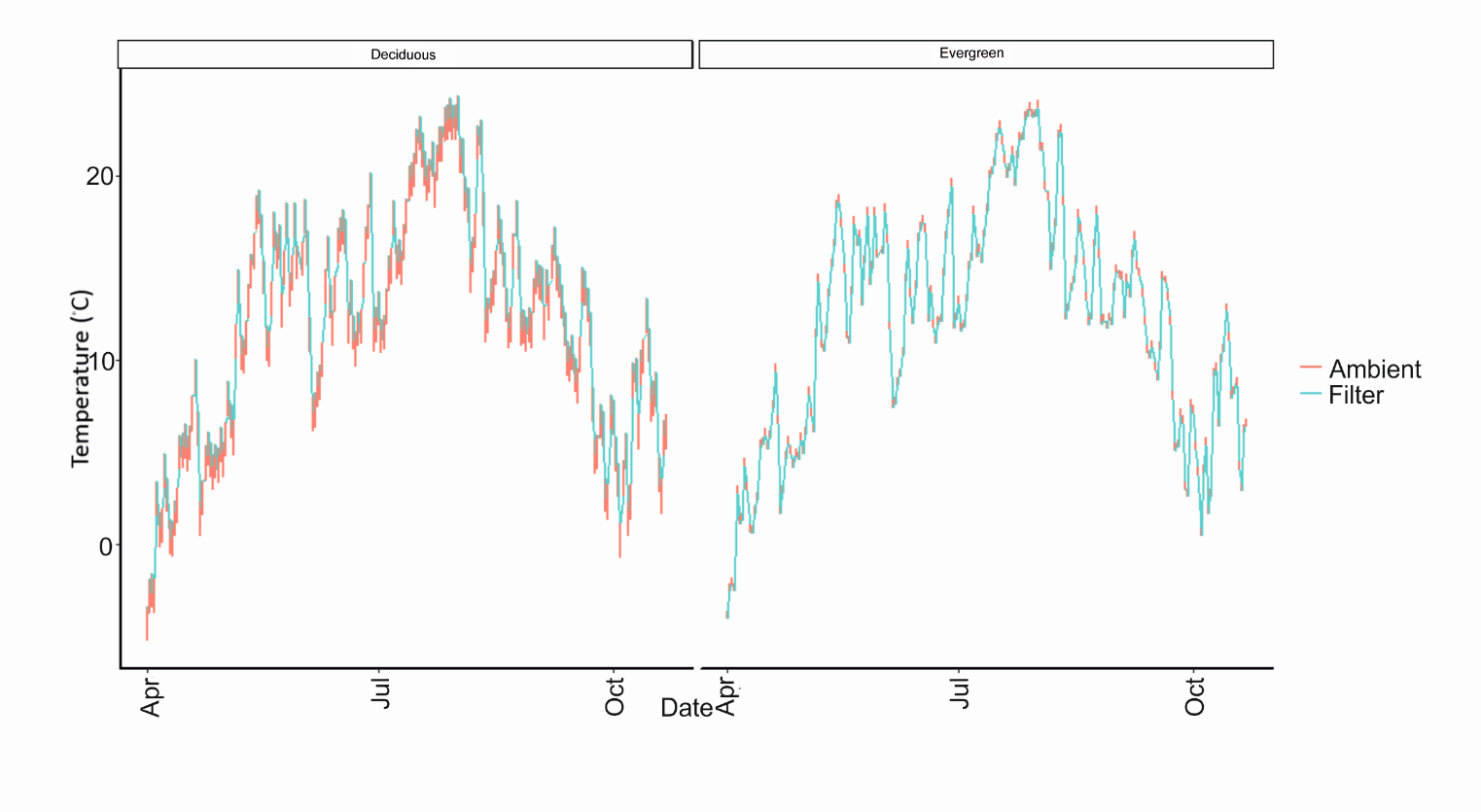


**Fig. S8**. Comparison of time-series temperature data for air temperature under the filters in the forest understorey and outside the stands (April to October 2018). Data from the FMI weather station outside the forest stands at Lammi Biological Station and in the forest understorey under the control filter treatment from the *Picea abies* stand (presented in Fig. S7).


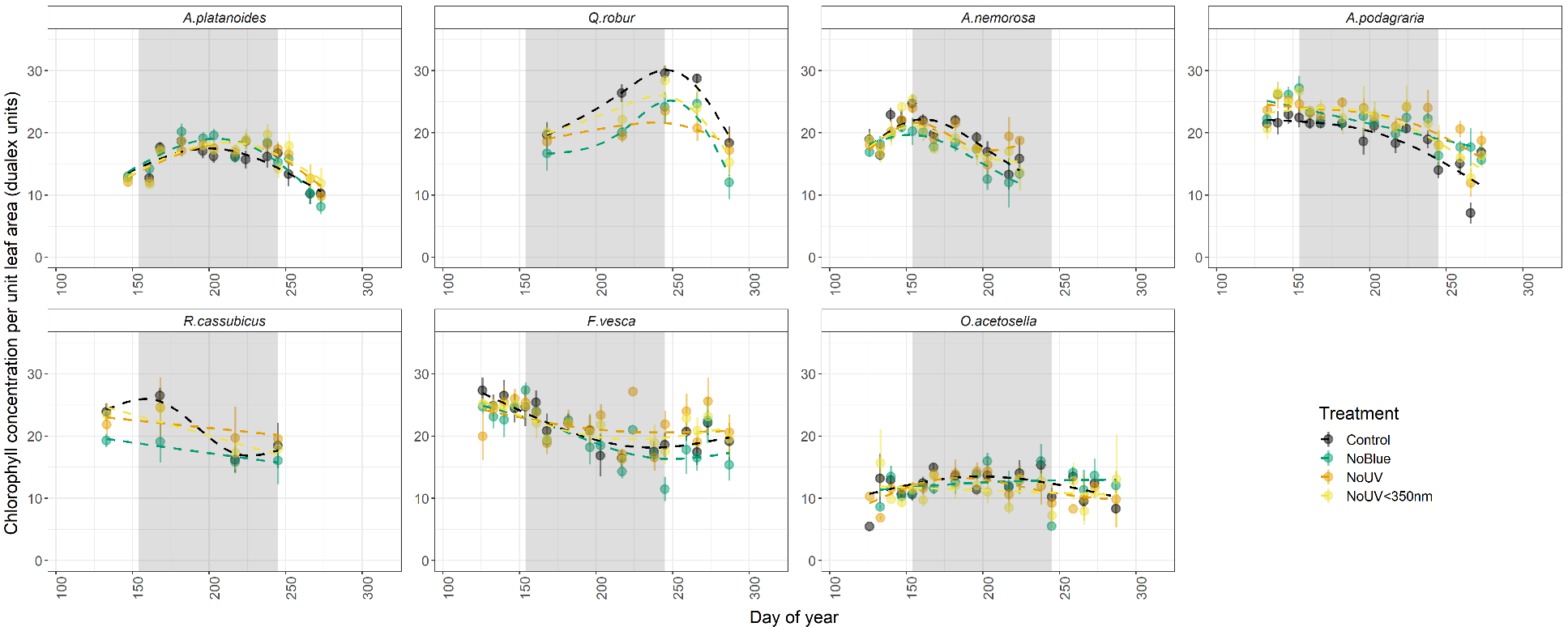


**Fig. S9A**. Leaf chlorophyll content (optical index – arbitrary units) of seven understorey plant species measured with a Dualex during 2017 and 2018, growing in deciduous stands. The grey shaded area represents the period when the canopy was closed. Means ± 1 SE presented on the graph with plot as the unit of replication.


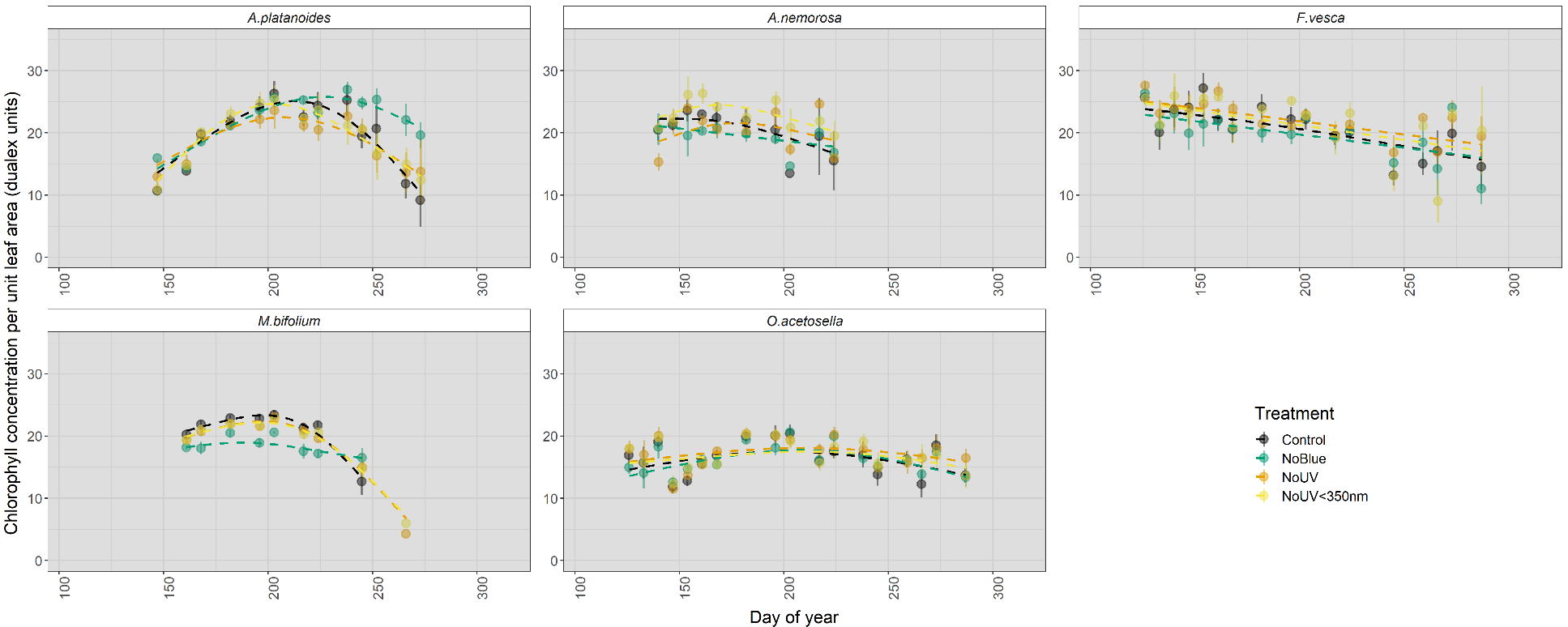


**Fig. S9B**. Leaf chlorophyll content (optical index – arbitrary units) of five understorey plant species measured with a Dualex during 2017 and 2018, growing in evergreen stands. The grey shaded area represents the period when the canopy was closed. Means ± 1 SE presented on the graph with plot as the unit of replication.


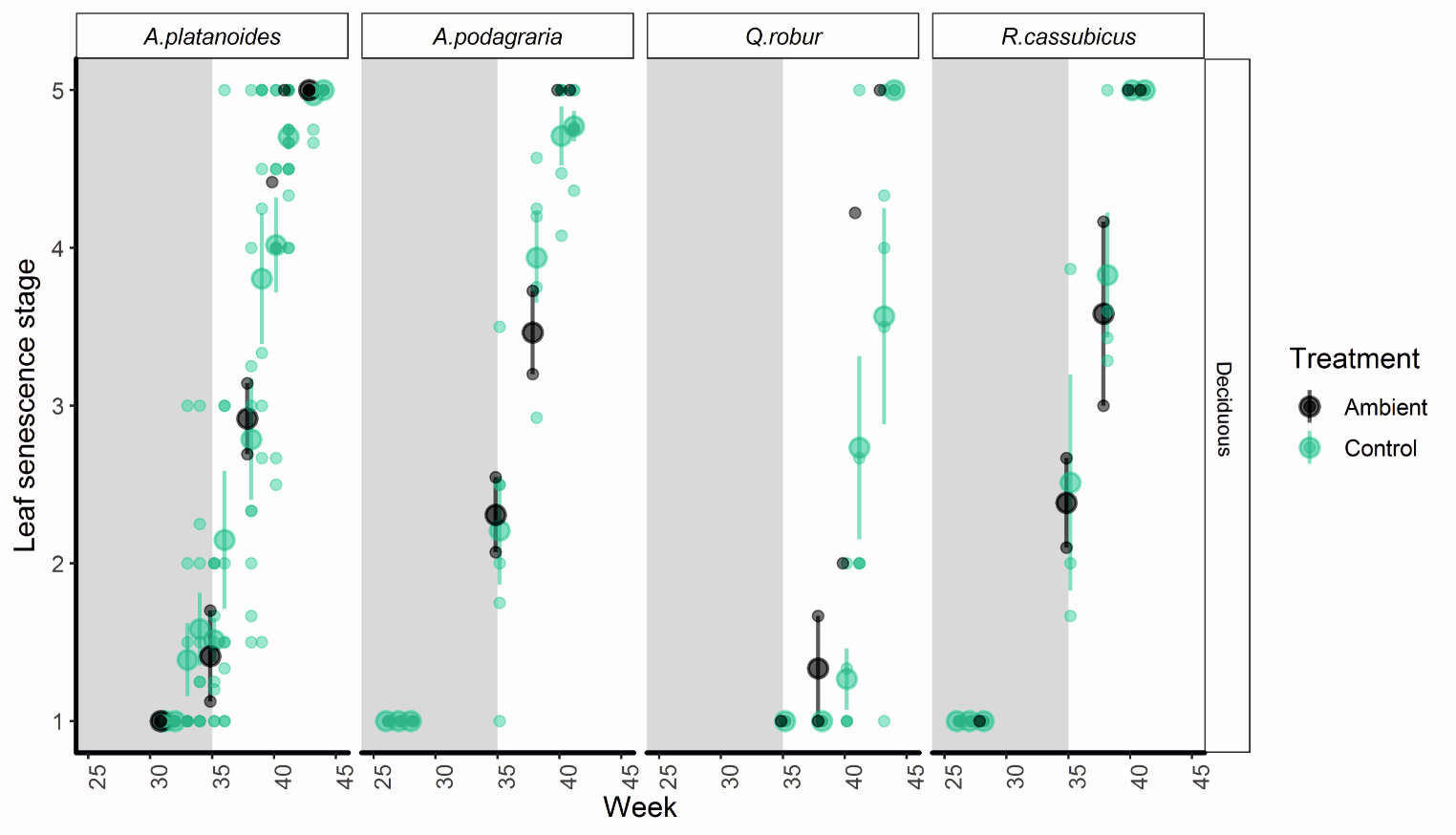


**Fig. S10**. Leaf senescence in autumn for different understorey species under a deciduous canopy in 2017 and 2018. Leaf senescence (scored on a 1-5 ordinal scale) under ambient conditions is shown in black and under the transparent control filters from plots in all deciduous stands in green. The grey shaded area represents the period when the canopy was closed and white following canopy leaf fall. Means ± 1 SE are presented with the points representing each plot (the unit of replication).

**Statistical Tables**

**Table S3A**. ANOVA output from GAMM on *A. platanoides* leaf out.

| ***A. platanoides* leaf out** |  |  |  |
| --- | --- | --- | --- |
|  | df | F | p-value |
| StandType | 1 | 11.699 | 0.000653 |
| Treatment | 3 | 10.544 | 8.04E-07 |
| StandType:Treatment | 3 | 1.732 | 0.158839 |

**Table S3B**. Summary output from GAMM on *A. platanoides* leaf out.

| ***A. platanoides* leaf out** |  |  |  |  |  |
| --- | --- | --- | --- | --- | --- |
|  | Estimate | Std. Error | t value | Pr(>\|t\|) |  |
| (Intercept) | 4.34737 | 0.07782 | 55.865 | < 2e-16 | *** |
| StandTypeEvergreen | -0.46859 | 0.137 | -3.42 | 0.000653 | *** |
| TreatmentNoBlue | -0.40546 | 0.08841 | -4.586 | 5.15E-06 | *** |
| TreatmentNoUV | 0.05505 | 0.08588 | 0.641 | 0.521693 |  |
| TreatmentNoUV<350nm | -0.15954 | 0.0866 | -1.842 | 0.065757 | . |
| StandTypeEvergreen:TreatmentNoBlue | 0.17632 | 0.15381 | 1.146 | 0.251948 |  |
| StandTypeEvergreen:TreatmentNoUV | -0.15013 | 0.15135 | -0.992 | 0.321489 |  |
| StandTypeEvergreen:TreatmentNoUV<350nm | 0.11243 | 0.1508 | 0.746 | 0.456118 |  |
| StandTypeEvergreen:TreatmentNoUV<350nm | 0.11243 | 0.1508 | 0.746 | 0.456118 |  |
|  |  |  |  |  |  |
|  | edf | Ref.df | F | p-value |  |
| s(Time) | 8.80E+00 | 8.797 | 657.42 | < 2e-16 | *** |
| ti(Time,Treatment) | 1.40E-06 | 12 | 0 | 0.603 |  |
| t2(YEAR,StandType,Stand,Time,Plot) | 1.11E+01 | 18 | 2.32 | 1.38E-06 | *** |
|  |  |  |  |  |  |
| **Comparison against UV filter** | Estimate | Std. Error | t value | Pr(>\|t\|) |  |
| (Intercept) | 4.39987 | 0.08064 | 54.565 | < 2e-16 | *** |
| StandTypeEvergreen | -0.62095 | 0.13632 | -4.555 | 5.94E-06 | *** |
| TreatmentControl | -0.05582 | 0.08605 | -0.649 | 0.5167 |  |
| TreatmentNoBlue | -0.46715 | 0.0899 | -5.196 | 2.50E-07 | *** |
| TreatmentNoUV<350nm | -0.21464 | 0.08792 | -2.441 | 0.0148 | * |
| StandTypeEvergreen:TreatmentControl | 0.15081 | 0.15166 | 0.994 | 0.3203 |  |
| StandTypeEvergreen:TreatmentNoBlue | 0.33327 | 0.15522 | 2.147 | 0.032 | * |
| StandTypeEvergreen:TreatmentNoUV<350nm | 0.2623 | 0.15209 | 1.725 | 0.0849 | . |

**Table S3C.** ANOVA output from GAMM on *A. nemorosa* leaf out.

| ***A. nemorosa* leaf out** |  |  |  |
| --- | --- | --- | --- |
|  | df | F | p-value |
| StandType | 1 | 9.049 | 0.003 |
| Treatment | 3 | 2.434 | 0.0664 |
| StandType:Treatment | 3 | 0.219 | 0.8829 |

**Table S3D**. Summary output from GAMM on *A. nemorosa* leaf out.

| ***A. nemorosa* leaf out** |  |  |  |  |  |
| --- | --- | --- | --- | --- | --- |
|  | Estimate | Std. Error | t value | Pr(>\|t\|) |  |
| (Intercept) | 1.88178 | 0.03861 | 48.732 | <2e-16 | *** |
| StandTypeEvergreen | -0.23393 | 0.07776 | -3.008 | 0.003 | ** |
| TreatmentNoBlue | -0.07353 | 0.05041 | -1.459 | 0.146 |  |
| TreatmentNoUV | -0.062 | 0.05277 | -1.175 | 0.242 |  |
| TreatmentNoUV<350nm | 0.05096 | 0.05155 | 0.989 | 0.324 |  |
| StandTypeEvergreen:TreatmentNoBlue | -0.06994 | 0.10801 | -0.648 | 0.518 |  |
| StandTypeEvergreen:TreatmentNoUV | 0.01555 | 0.10725 | 0.145 | 0.885 |  |
| StandTypeEvergreen:TreatmentNoUV<350nm | -0.01199 | 0.10447 | -0.115 | 0.909 |  |
|  |  |  |  |  |  |
|  | edf | Ref.df | F | p-value |  |
| s(Time) | 2.609 | 2.609 | 90.12 | <2e-16 | *** |
| t2(Time,StandType,Stand,Plot) | 4.007 | 13 | 0.58 | 0.0718 | . |

**Table S3E**. ANOVA output from GAMM on *A. podagraria* leaf out.

| ***A. podagraria* leaf out** |  |  |  |
| --- | --- | --- | --- |
|  | df | F | p-value |
| Treatment | 3 | 1.816 | 0.144 |

**Table S3F**. ANOVA output from GAMM on *R. cassubicus* leaf out.

| ***R. cassubicus* leaf out** |  |  |  |
| --- | --- | --- | --- |
|  | df | F | p-value |
| Treatment | 3 | 0.924 | 0.43 |

**Table S4A**. ANOVA output from GAMM on *A. platanoides* leaf senescence.

| ***A. platanoides* senescence** |  |  |  |  |
| --- | --- | --- | --- | --- |
|  | df | F | p-value |  |
| StandType | 1 | 0.008 | 0.929763 |  |
| Treatment | 3 | 8.611 | 1.24E-05 |  |
| StandType:Treatment | 3 | 6.684 | 0.000184 |  |

**Table S4B**. Summary output from GAMM on *A. platanoides* leaf senescence.

| ***A. platanoides* senescence** |  |  |  |  |  |
| --- | --- | --- | --- | --- | --- |
|  | Estimate | Std. Error | t value | Pr(>\|t\|) |  |
| (Intercept) | 2.95749 | 0.07938 | 37.258 | < 2e-16 | *** |
| StandTypeEvergreen | -0.01102 | 0.12497 | -0.088 | 0.92976 |  |
| TreatmentNoBlue | -0.40981 | 0.0825 | -4.968 | 8.20E-07 | *** |
| TreatmentNoUV | -0.26135 | 0.08214 | -3.182 | 0.00152 | ** |
| TreatmentNoUV<350nm | -0.26527 | 0.08167 | -3.248 | 0.00121 | ** |
| StandTypeEvergreen:TreatmentNoBlue | -0.35335 | 0.13373 | -2.642 | 0.00839 | ** |
| StandTypeEvergreen:TreatmentNoUV | 0.21916 | 0.12966 | 1.69 | 0.09133 | . |
| StandTypeEvergreen:TreatmentNoUV<350nm | 0.09923 | 0.12936 | 0.767 | 0.44325 |  |
|  |  |  |  |  |  |
|  | edf | Ref.df | F | p-value |  |
| s(Time) | 6.34 | 6.34 | 219.09 | < 2e-16 | *** |
| ti(Time,Treatment) | 7.213 | 12 | 3.703 | 2.09E-08 | *** |
| t2(YEAR,StandType,Stand,Time,Plot) | 10.199 | 15 | 3.581 | 5.47E-09 | *** |

| **Comparison against UV filter** |  |  |  |  |  |
| --- | --- | --- | --- | --- | --- |
|  | Estimate | Std. Error | t value | Pr(>\|t\|) |  |
| (Intercept) | 2.6967 | 0.08047 | 33.511 | < 2e-16 | *** |
| StandTypeEvergreen | 0.20584 | 0.12601 | 1.633 | 0.10274 |  |
| TreatmentControl | 0.26309 | 0.08424 | 3.123 | 0.00185 | ** |
| TreatmentNoBlue | -0.14413 | 0.08508 | -1.694 | 0.09062 | . |
| TreatmentNoUV<350nm | -0.00321 | 0.08424 | -0.038 | 0.96961 |  |
| StandTypeEvergreen:TreatmentControl | -0.21772 | 0.13297 | -1.637 | 0.10192 |  |
| StandTypeEvergreen:TreatmentNoBlue | -0.57265 | 0.13719 | -4.174 | 3.30E-05 | *** |
| StandTypeEvergreen:TreatmentNoUV<350nm | -0.12086 | 0.13274 | -0.911 | 0.36281 |  |

**Table S4C**. ANOVA output from GAMM on *R. cassubicus* leaf senescence.

| ***R. cassubicus* senescence** |  |  |  |
| --- | --- | --- | --- |
|  | df | F | p-value |
| Treatment | 3 | 3.595 | 0.0164 |

**Table S4D.** Summary output from GAMM on *R. cassubicus* leaf senescence

| ***R. cassubicus* senescence** |  |  |  |  |  |
| --- | --- | --- | --- | --- | --- |
|  | Estimate | Std. Error | t value | Pr(>\|t\|) |  |
| (Intercept) | 2.49253 | 0.08389 | 29.711 | < 2e-16 | *** |
| TreatmentNoBlue | -0.44614 | 0.1372 | -3.252 | 0.00158 | ** |
| TreatmentNoUV | -0.11893 | 0.13659 | -0.871 | 0.38605 |  |
| TreatmentNoUV<350nm | -0.18053 | 0.12055 | -1.498 | 0.1375 |  |
|  | edf | Ref.df | F | p-value |  |
| s(Time) | 3.31E+00 | 3.309 | 340.5 | <2e-16 | *** |
| t2(Time,Stand,Plot) | 2.44E-07 | 5 | 0 | 0.374 |  |
| **Comparison against UV filter** |  |  |  |  |  |
|  | Estimate | Std. Error | t value | Pr(>\|t\|) |  |
| (Intercept) | 2.3736 | 0.1078 | 22.015 | <2e-16 | *** |
| TreatmentControl | 0.1189 | 0.1366 | 0.871 | 0.386 |  |
| TreatmentNoBlue | -0.3272 | 0.1532 | -2.136 | 0.0352 | * |
| TreatmentNoUV<350nm | -0.0616 | 0.1382 | -0.446 | 0.6568 |  |

**Table S4E.** ANOVA output from GAMM on *A. podagraria* leaf senescence.

| ***A. podagraria* senescence** |  |  |  |
| --- | --- | --- | --- |
|  | df | F | p-value |
| Treatment | 3 | 3.848 | 0.0114 |

**Table S4F.** Summary output from GAMM on *A. podagraria* leaf senescence.

| ***A. podagraria* senescence** |  |  |  |  |  |
| --- | --- | --- | --- | --- | --- |
|  | Estimate | Std. Error | t value | Pr(>\|t\|) |  |
| (Intercept) | 2.65401 | 0.10321 | 25.714 | < 2e-16 | *** |
| TreatmentNoBlue | -0.33591 | 0.11478 | -2.927 | 0.00412 | ** |
| TreatmentNoUV | -0.27243 | 0.11177 | -2.437 | 0.01631 | * |
| TreatmentNoUV<350nm | -0.07927 | 0.11317 | -0.7 | 0.48507 |  |

|  | edf | Ref.df | F | p-value |  |
| --- | --- | --- | --- | --- | --- |
| s(Time) | 3.577 | 3.577 | 362.146 | <2e-16 | *** |
| t2(Time,Stand,Plot) | 3.421 | 5 | 4.628 | 8.00E-05 | *** |
|  |  |  |  |  |  |
| **Comparison against UV filter** |  |  |  |  |  |
|  | Estimate | Std. Error | t value | Pr(>\|t\|) |  |
| (Intercept) | 2.38158 | 0.11347 | 20.988 | <2e-16 | *** |
| TreatmentControl | 0.27243 | 0.11177 | 2.437 | 0.0163 | * |
| TreatmentNoBlue | -0.06348 | 0.11649 | -0.545 | 0.5869 |  |
| TreatmentNoUV<350nm | 0.19317 | 0.11469 | 1.684 | 0.0948 | . |

**Table S4G.** ANOVA output from GAMM on *Q. robur* leaf senescence.

| ***Q. robur* senescence** |  |  |  |
| --- | --- | --- | --- |
|  | df | F | p-value |
| Treatment | 3 | 2.189 | 0.0949 |

| ***A. platanoides* adaxial flavonols** |  |  |  |
| --- | --- | --- | --- |
|  | df | F | p-value |
| StandType | 1 | 94.39 | <2e-16 |
| Treatment | 3 | 205.66 | <2e-16 |
| StandType:Treatment | 3 | 30.9 | <2e-16 |

**Table S5A**. ANOVA output from GAMM on *A. platanoides* adaxial flavonols.

**Table S5B**. Summary output from GAMM on *A. platanoides* adaxial flavonols.

| ***A. platanoides* adaxial flavonol** |  |  |  |  |  |
| --- | --- | --- | --- | --- | --- |
|  | Estimate | Std. Error | t value | Pr(>\|t\|) |  |
| (Intercept) | 0.9024 | 0.033401 | 27.017 | < 2e-16 | *** |
| StandTypeEvergreen | -0.45618 | 0.046954 | -9.716 | < 2e-16 | *** |
| TreatmentNoBlue | -0.43314 | 0.017849 | -24.266 | < 2e-16 | *** |
| TreatmentNoUV | -0.12648 | 0.017401 | -7.269 | 9.14E-13 | *** |
| TreatmentNoUV<350nm | -0.17045 | 0.017456 | -9.765 | < 2e-16 | *** |
| StandTypeEvergreen:TreatmentNoBlue | 0.248783 | 0.029508 | 8.431 | < 2e-16 | *** |
| StandTypeEvergreen:TreatmentNoUV | 0.004177 | 0.028484 | 0.147 | 0.8835 |  |
| StandTypeEvergreen:TreatmentNoUV<350nm | 0.046779 | 0.02811 | 1.664 | 0.0965 | . |
|  | edf | Ref.df | F | p-value |  |
| s(Week) | 8.183 | 8.183 | 5.36E+00 | 1.78E-06 |  |
| ti(Week,Treatment) | 12 | 12 | 0 | 1 |  |
| t2(YEAR,Week,StandType,Stand,PLOT) | 15.862 | 18 | 22.417 | < 2e-16 |  |
|  |  |  |  |  |  |
| **Comparison against UV filter** |  |  |  |  |  |
|  | Estimate | Std. Error | t value | Pr(>\|t\|) |  |
| (Intercept) | 0.775918 | 0.033454 | 23.194 | < 2e-16 | *** |
| StandTypeEvergreen | -0.452 | 0.047244 | -9.568 | < 2e-16 | *** |
| TreatmentControl | 0.126481 | 0.017401 | 7.269 | 9.14E-13 | *** |
| TreatmentNoBlue | -0.30665 | 0.017829 | -17.199 | < 2e-16 | *** |
| TreatmentNoUV<350nm | -0.04397 | 0.017476 | -2.516 | 0.0121 | * |
| StandTypeEvergreen:TreatmentControl | -0.00418 | 0.028484 | -0.147 | 0.8835 |  |
| StandTypeEvergreen:TreatmentNoBlue | 0.244607 | 0.029534 | 8.282 | 5.56E-16 | *** |
| StandTypeEvergreen:TreatmentNoUV<350nm | 0.042603 | 0.028386 | 1.501 | 0.1338 |  |

**Table S5C**. ANOVA output from GAMM on *A. podagraria* adaxial flavonols.

| ***A. podagraria* adaxial flavonols** |  |  |  |
| --- | --- | --- | --- |
|  | df | F | p-value |
| Treatment | 3 | 71.64 | <2e-16 |

**Table S5D**. Summary output from GAMM on *A. podagraria* adaxial flavonols.

| ***A. podagraria* adaxial flavonols** |  |  |  |  |  |
| --- | --- | --- | --- | --- | --- |
|  |  |  |  |  |  |
|  | Estimate | Std. Error | t value | Pr(>\|t\|) |  |
| (Intercept) | 0.74681 | 0.07024 | 10.632 | <2e-16 | *** |
| TreatmentNoBlue | -0.31161 | 0.02476 | -12.586 | <2e-16 | *** |
| TreatmentNoUV | -0.05332 | 0.02544 | -2.096 | 0.037 | * |
| TreatmentNoUV<350nm | -0.05287 | 0.02793 | -1.893 | 0.0594 | . |
|  |  |  |  |  |  |
|  |  |  |  |  |  |
|  | edf | Ref.df | F | p-value |  |
| s(Week):TreatmentControl | 5.126 | 5.126 | 6.62 | 9.54E-06 | *** |
| s(Week):TreatmentNoBlue | 1 | 1 | 1.4 | 0.237699 |  |
| s(Week):TreatmentNoUV | 3.507 | 3.507 | 4.07 | 0.003236 | ** |
| s(Week):TreatmentNoUV<350nm | 4.354 | 4.354 | 5.837 | 0.000174 | *** |
| ti(Week,Treatment) | 4.856 | 12 | 3.026 | 2.06E-08 | *** |
| t2(YEAR,Week,Stand,PLOT) | 6.82 | 9 | 12.015 | < 2e-16 | *** |
|  |  |  |  |  |  |
| **Comparison against UV filter** |  |  |  |  |  |
|  | Estimate | Std. Error | t value | Pr(>\|t\|) |  |
| (Intercept) | 0.720174 | 0.048602 | 14.818 | <2e-16 | *** |
| TreatmentControl | 0.046217 | 0.027805 | 1.662 | 0.0976 | . |
| TreatmentNoBlue | -0.26582 | 0.026666 | -9.968 | <2e-16 | *** |
| TreatmentNoUV<350nm | -0.00344 | 0.028985 | -0.119 | 0.9057 |  |

**Table S5E**. ANOVA output from LME on *A. nemorosa* adaxial flavonols.

| ***A. nemorosa* adaxial flavonols** |  |  |  |
| --- | --- | --- | --- |
| Parametric | Terms: |  |  |
|  | df | F | p-value |
| StandType | 1.00 | 209.90 | <2e-16 |
| Treatment | 3.00 | 252.26 | <2e-16 |
| StandType:Treatment | 3.00 | 35.15 | <2e-16 |

**Table S5F**. Summary output from LME on *A. nemorosa* adaxial flavonols.

| ***A. nemerosa* adaxial flavonols** |  |  |  |  |  |
| --- | --- | --- | --- | --- | --- |
|  | Estimate | Std. Error | t value | Pr(>\|t\|) |  |
| (Intercept) | 1.07667 | 0.02438 | 44.169 | < 2e-16 | *** |
| StandTypeEvergreen | -0.63778 | 0.04901 | -13.013 | < 2e-16 | *** |
| TreatmentNoBlue | -0.47333 | 0.01864 | -25.394 | < 2e-16 | *** |
| TreatmentNoUV | -0.13988 | 0.0181 | -7.73 | 6.72E-14 | *** |
| TreatmentNoUV<350nm | -0.09258 | 0.0177 | -5.232 | 2.54E-07 | *** |
| StandTypeEvergreen:TreatmentNoBlue | 0.36708 | 0.04124 | 8.902 | < 2e-16 | *** |
| StandTypeEvergreen:TreatmentNoUV | 0.04004 | 0.03708 | 1.08 | 0.2808 |  |
| StandTypeEvergreen:TreatmentNoUV<350nm | 0.06765 | 0.03735 | 1.811 | 0.0707 | . |
|  |  |  |  |  |  |
|  |  |  |  |  |  |
|  | edf | Ref.df | F | p-value |  |
| s(Week) | 7.264 | 7.264 | 20.328 | < 2e-16 | *** |
| ti(Week,Treatment) | 4.924 | 12 | 1.177 | 0.00482 | ** |
| t2(Week,StandType,Stand,REALPLOT) | 10.823 | 15 | 7.557 | < 2e-16 | *** |
| **Comparison against UV filter** |  |  |  |  |  |
|  | Estimate | Std. Error | t value | Pr(>\|t\|) |  |
| (Intercept) | 0.93678 | 0.02456 | 38.148 | < 2e-16 | *** |
| StandTypeEvergreen | -0.59774 | 0.04747 | -12.592 | < 2e-16 | *** |
| TreatmentControl | 0.13988 | 0.0181 | 7.73 | 6.72E-14 | *** |
| TreatmentNoBlue | -0.33345 | 0.01895 | -17.599 | < 2e-16 | *** |
| TreatmentNoUV<350nm | 0.0473 | 0.01814 | 2.608 | 0.00941 | ** |
| StandTypeEvergreen:TreatmentControl | -0.04004 | 0.03708 | -1.08 | 0.28079 |  |
| StandTypeEvergreen:TreatmentNoBlue | 0.32703 | 0.04063 | 8.049 | 7.08E-15 | *** |
| StandTypeEvergreen:TreatmentNoUV<350nm | 0.02761 | 0.03676 | 0.751 | 0.45303 |  |

**Table S5G**. ANOVA output from GAMM on *F. vesca* adaxial flavonols.

| ***F. vesca* adaxial flavonols** |  |  |  |
| --- | --- | --- | --- |
|  |  |  |  |
|  | df | F | p-value |
| StandType | 1 | 237.78 | < 2e-16 |
| Treatment | 3 | 12.51 | 5.42E-08 |
| StandType:Treatment | 3 | 1.04 | 0.374 |

**Table S5H**. Summary output from GAMM on *F. vesca* adaxial flavonols.

| ***F. vesca* adaxial flavonols** |  |  |  |  |  |
| --- | --- | --- | --- | --- | --- |
|  |  |  |  |  |  |
|  | Estimate | Std. Error | t value | Pr(>\|t\|) |  |
| (Intercept) | 0.924708 | 0.085554 | 10.808 | < 2e-16 | *** |
| StandTypeEvergreen | -0.95907 | 0.062196 | -15.42 | < 2e-16 | *** |
| TreatmentNoBlue | -0.16252 | 0.028204 | -5.762 | 1.20E-08 | *** |
| TreatmentNoUV | -0.13418 | 0.027362 | -4.904 | 1.14E-06 | *** |
| TreatmentNoUV<350nm | -0.08496 | 0.02806 | -3.028 | 0.00254 | ** |
| StandTypeEvergreen:TreatmentNoBlue | -0.00332 | 0.036372 | -0.091 | 0.9272 |  |
| StandTypeEvergreen:TreatmentNoUV | 0.045374 | 0.036063 | 1.258 | 0.20869 |  |
| StandTypeEvergreen:TreatmentNoUV<350nm | -0.00733 | 0.037981 | -0.193 | 0.84701 |  |
|  |  |  |  |  |  |
|  | edf | Ref.df | F | p-value |  |
| s(Week) | 6.981 | 6.981 | 32.414 | < 2e-16 | *** |
| ti(Week,Treatment) | 4.669 | 12 | 1.271 | 0.00134 | ** |
| t2(YEAR,Week,StandType,Stand,PLOT) | 18.693 | 21 | 11.717 | < 2e-16 | *** |
|  |  |  |  |  |  |
| **Comparison against UV filter** |  |  |  |  |  |
|  | Estimate | Std. Error | t value | Pr(>\|t\|) |  |
| (Intercept) | 0.955875 | 0.115894 | 8.248 | 6.87E-16 | *** |
| StandTypeEvergreen | -1.28688 | 0.071766 | -17.932 | < 2e-16 | *** |
| TreatmentControl | 0.100458 | 0.029989 | 3.35 | 0.000848 | *** |
| TreatmentNoBlue | -0.03501 | 0.03015 | -1.161 | 0.245897 |  |
| TreatmentNoUV<350nm | 0.027881 | 0.030545 | 0.913 | 0.361629 |  |
| StandTypeEvergreen:TreatmentControl | -0.00271 | 0.043483 | -0.062 | 0.950299 |  |
| StandTypeEvergreen:TreatmentNoBlue | -0.03284 | 0.043833 | -0.749 | 0.453994 |  |
| StandTypeEvergreen:TreatmentNoUV<350nm | -0.03105 | 0.045631 | -0.68 | 0.49644 |  |

**Table S5I.** ANOVA output from GAMM on *M. bifolium* adaxial flavonols.

| ***M. bifolium* adaxial flavonols** |  |  |  |
| --- | --- | --- | --- |
|  |  |  |  |
|  | df | F | p-value |
| Treatment | 3 | 23.22 | 1.42E-12 |

**Table S5J**. Summary output from GAMM on *M. bifolium* adaxial flavonols.

| ***M.bifolium* adaxial flavonols** |  |  |  |  |  |
| --- | --- | --- | --- | --- | --- |
|  |  |  |  |  |  |
|  | Estimate | Std. Error | t value | Pr(>\|t\|) |  |
| (Intercept) | 0.282182 | 0.005917 | 47.69 | < 2e-16 | *** |
| TreatmentNoBlue | -0.05069 | 0.006276 | -8.077 | 1.37E-13 | *** |
| TreatmentNoUV | -0.02296 | 0.005943 | -3.863 | 0.000161 | *** |
| TreatmentNoUV<350nm | -0.01718 | 0.006457 | -2.661 | 0.008573 | ** |
|  |  |  |  |  |  |
|  | edf | Ref.df | F | p-value |  |
| s(Week) | 3.683 | 3.683 | 17.886 | 7.73E-12 | *** |
| ti(Week,Treatment) | 2.875 | 12 | 0.449 | 0.133005 |  |
| t2(YEAR,Week,PLOT) | 3.683 | 6 | 3.234 | 0.000262 | *** |
|  |  |  |  |  |  |
| **Comparison against UV filter** |  |  |  |  |  |
|  | Estimate | Std. Error | t value | Pr(>\|t\|) |  |
| (Intercept) | 0.266396 | 0.005752 | 46.313 | < 2e-16 | *** |
| TreatmentControl | 0.041521 | 0.007565 | 5.488 | 1.51E-07 | *** |
| TreatmentNoBlue | -0.02177 | 0.008629 | -2.523 | 0.0126 | * |
| TreatmentNoUV<350nm | 0.004228 | 0.008288 | 0.51 | 0.6107 |  |

**Table S5K**. ANOVA output from GAMM on *O. acetosella* adaxial flavonols.

| ***O. acetosella* adaxial flavonols** |  |  |  |
| --- | --- | --- | --- |
|  |  |  |  |
|  | df | F | p-value |
| StandType | 1 | 175.25 | < 2e-16 |
| Treatment | 3 | 26 | 7.44E-16 |
| StandType:Treatment | 3 | 11.54 | 2.26E-07 |

**Table S5L.** Summary output from GAMM on *O. acetosella* adaxial flavonols.

| ***O. acetosella* adaxial flavonols** |  |  |  |  |  |
| --- | --- | --- | --- | --- | --- |
|  |  |  |  |  |  |
|  | Estimate | Std. Error | t value | Pr(>\|t\|) |  |
| (Intercept) | 0.73618 | 0.03249 | 22.659 | < 2e-16 | *** |
| StandTypeEvergreen | -0.36534 | 0.0276 | -13.238 | < 2e-16 | *** |
| TreatmentNoBlue | -0.12901 | 0.01548 | -8.336 | 4.92E-16 | *** |
| TreatmentNoUV | -0.03908 | 0.01529 | -2.555 | 0.010861 | * |
| TreatmentNoUV<350nm | -0.0216 | 0.01532 | -1.409 | 0.159254 |  |
| StandTypeEvergreen:TreatmentNoBlue | -0.02763 | 0.0178 | -1.552 | 0.121207 |  |
| StandTypeEvergreen:TreatmentNoUV | -0.09662 | 0.01767 | -5.469 | 6.56E-08 | *** |
| StandTypeEvergreen:TreatmentNoUV<350nm | -0.06723 | 0.01786 | -3.764 | 0.000183 | *** |
|  |  |  |  |  |  |
|  | edf | Ref.df | F | p-value |  |
| s(Week) | 7.462 | 7.462 | 48.121 | < 2e-16 | *** |
| ti(Week,Treatment) | 2.478 | 12 | 2.811 | 4.20E-08 | *** |
| t2(YEAR,Week,StandType,Stand,PLOT) | 11.94 | 14 | 9.771 | < 2e-16 | *** |
|  |  |  |  |  |  |
| **Comparison against UV filter** |  |  |  |  |  |
|  | Estimate | Std. Error | t value | Pr(>\|t\|) |  |
| (Intercept) | 0.70281 | 0.03244 | 21.667 | < 2e-16 | *** |
| StandTypeEvergreen | -0.4601 | 0.02686 | -17.133 | < 2e-16 | *** |
| TreatmentControl | 0.04176 | 0.01374 | 3.039 | 0.002471 | ** |
| TreatmentNoBlue | -0.08862 | 0.01506 | -5.883 | 6.56E-09 | *** |
| TreatmentNoUV<350nm | 0.01839 | 0.01408 | 1.306 | 0.191948 |  |
| StandTypeEvergreen:TreatmentControl | 0.10363 | 0.01684 | 6.156 | 1.34E-09 | *** |
| StandTypeEvergreen:TreatmentNoBlue | 0.06863 | 0.01796 | 3.822 | 0.000145 | *** |
| StandTypeEvergreen:TreatmentNoUV<350nm | 0.03288 | 0.01712 | 1.92 | 0.055294 | . |

**Table S5M.** ANOVA output from GAMM on *Q. robur* adaxial flavonols.

| ***Q. robur* adaxial flavonols** | Terms: |  |  |
| --- | --- | --- | --- |
|  | df | F | p-value |
| Treatment | 3 | 19.89 | 1.04E-09 |

**Table S5N**. Summary output from GAMM on *Q. robur* adaxial flavonols.

| ***Q. robur* adaxial flavonols** |  |  |  |  |  |
| --- | --- | --- | --- | --- | --- |
|  |  |  |  |  |  |
|  | Estimate | Std.Error | t-value | p-value |  |
| (Intercept) | 0.61733 | 0.03405 | 18.132 | < 2e-16 | *** |
| TreatmentNoBlue | -0.27244 | 0.03577 | -7.616 | 3.33E-11 | *** |
| TreatmentNoUV | -0.2455 | 0.03661 | -6.706 | 2.08E-09 | *** |
| TreatmentNoUV>350nm | -0.17692 | 0.04001 | -4.422 | 2.88E-05 | *** |
| --- |  |  |  |  |  |
|  | edf | Ref.df | F | p-value |  |
| s(Week):TreatmentControl | 1 | 1 | 20.653 | 1.71E-05 | *** |
| s(Week):TreatmentNoBlue | 1 | 1 | 7.487 | 0.00753 | ** |
| s(Week):TreatmentNoUV | 1 | 1 | 2.639 | 0.10794 |  |
| s(Week):TreatmentNoUV>350nm | 1 | 1 | 2.792 | 0.09838 | . |
| t2(Week,Stand,REALPLOT) | 4.384 | 7 | 4.364 | 3.29E-05 | *** |
|  |  |  |  |  |  |
| **Comparison against UV filter** |  |  |  |  |  |
|  |  |  |  |  |  |
|  | Estimate | Std. Error | t value | Pr(>\|t\|) |  |
| (Intercept) | 0.37334 | 0.03986 | 9.367 | 5.20E-14 | *** |
| TreatmentControl | 0.24197 | 0.039 | 6.205 | 3.31E-08 | *** |
| TreatmentNoBlue | -0.02019 | 0.03882 | -0.52 | 0.6045 |  |
| TreatmentNoUV<350nm | 0.0737 | 0.04226 | 1.744 | 0.0855 | . |

**Table S5O.** ANOVA output from GAMM on *R. cassubicus* adaxial flavonols.

| ***R. cassubicus* adaxial flavonols** | df | F | p-value | p-value |
| --- | --- | --- | --- | --- |
| Treatment | 3 | 11.98 | 5.80E-06 | <.0001 |

**Table S5P.** Summary output from GAMM on *R. cassubicus* adaxial flavonols.

| ***R. cassubicus* adaxial flavonols** |  |  |  |  |  |
| --- | --- | --- | --- | --- | --- |
|  |  |  |  |  |  |
|  | Value | Std.Error | DF | t-value | p-value |
| (Intercept) | 0.8375563 | 0.138761 | 54 | 6.035975 | 0 |
| Week | -0.0055535 | 0.005304 | 54 | -1.04695 | 0.2998 |
| TreatmentNoBlue | -0.5109987 | 0.213684 | 54 | -2.39138 | 0.0203 |
| TreatmentNoUV | -0.0583252 | 0.215603 | 54 | -0.27052 | 0.7878 |
| TreatmentNoUV>350nm | -0.0201342 | 0.177674 | 54 | -0.11332 | 0.9102 |
| Week:TreatmentNoBlue | 0.0075988 | 0.008037 | 54 | 0.945491 | 0.3486 |
| Week:TreatmentNoUV | -0.0038703 | 0.008831 | 54 | -0.43826 | 0.6629 |
| Week:TreatmentNoUV>350nm | -0.0036133 | 0.00688 | 54 | -0.52521 | 0.6016 |
|  |  |  |  |  |  |
|  | edf | Ref.df | F | p-value |  |
| s(Week) | 2.031 | 2.031 | 3.511 | 0.04482 | * |
| t2(Week,REALPLOT) | 3.191 | 5 | 3.163 | 0.00363 | ** |
|  |  |  |  |  |  |
| **Comparison against UV filter** |  |  |  |  |  |
|  | Estimate | Std. Error | t value | Pr(>\|t\|) |  |
| (Intercept) | 0.56925 | 0.05334 | 10.671 | 4.01E-14 | *** |
| TreatmentControl | 0.15212 | 0.0557 | 2.731 | 0.00887 | ** |
| TreatmentNoBlue | -0.17831 | 0.06192 | -2.879 | 0.00599 | ** |
| TreatmentNoUV<350nm | 0.04168 | 0.0546 | 0.763 | 0.44906 |  |

**Table S6A**. ANOVA output from GAMM on *A. platanoides* adaxial anthocyanins.

| ***A. platanoides* adaxial anthocyanins** |  |  |  |
| --- | --- | --- | --- |
|  |  |  |  |
|  | df | F | p-value |
| StandType | 1 | 16.718 | 4.79E-05 |
| Treatment | 3 | 8.673 | 1.15E-05 |
| StandType:Treatment | 3 | 1.076 | 0.359 |

**Table S6B**. Summary output from GAMM on *A. platanoides* adaxial anthocyanins.

| ***A. platanoides* adaxial Anth** |  |  |  |  |  |
| --- | --- | --- | --- | --- | --- |
|  |  |  |  |  |  |
|  | Estimate | Std. Error | t value | Pr(>\|t\|) |  |
| (Intercept) | 0.430039 | 0.007289 | 58.999 | < 2e-16 | *** |
| StandTypeEvergreen | -0.04735 | 0.011581 | -4.089 | 4.79E-05 | *** |
| TreatmentNoBlue | -0.03395 | 0.007017 | -4.837 | 1.59E-06 | *** |
| TreatmentNoUV | -0.01472 | 0.006924 | -2.126 | 0.03379 | * |
| TreatmentNoUV<350nm | -0.02498 | 0.00691 | -3.615 | 0.00032 | *** |
| StandTypeEvergreen:TreatmentNoBlue | -0.00451 | 0.0111 | -0.406 | 0.68487 |  |
| StandTypeEvergreen:TreatmentNoUV | 0.009915 | 0.010938 | 0.906 | 0.36497 |  |
| StandTypeEvergreen:TreatmentNoUV<350nm | 0.012416 | 0.010714 | 1.159 | 0.24687 |  |
|  |  |  |  |  |  |
|  |  |  |  |  |  |
|  | edf | Ref.df | F | p-value |  |
| s(Week) | 6.8118 | 6.812 | 63.804 | < 2e-16 | *** |
| ti(Week,Treatment) | 0.9165 | 12 | 0.122 | 0.208 |  |
| t2(YEAR,Week,StandType,Stand,PLOT) | 13.066 | 18 | 5.161 | 3.81E-15 | *** |
|  |  |  |  |  |  |
| **Comparison against UV filter** |  |  |  |  |  |
|  | Estimate | Std. Error | t value | Pr(>\|t\|) |  |
| (Intercept) | 0.181391 | 0.006903 | 26.277 | <2e-16 | *** |
| StandTypeEvergreen | -0.02368 | 0.011361 | -2.084 | 0.0375 | * |
| TreatmentControl | 0.017295 | 0.006802 | 2.543 | 0.0112 | * |
| TreatmentNoBlue | -0.01669 | 0.006997 | -2.386 | 0.0173 | * |
| TreatmentNoUV<350nm | -0.00398 | 0.006851 | -0.581 | 0.5613 |  |
| StandTypeEvergreen:TreatmentControl | -0.00589 | 0.011109 | -0.53 | 0.596 |  |
| StandTypeEvergreen:TreatmentNoBlue | -0.02153 | 0.011538 | -1.866 | 0.0624 | . |
| StandTypeEvergreen:TreatmentNoUV<350nm | -0.00579 | 0.011074 | -0.523 | 0.6013 |  |

**Table S6C**. ANOVA output from GAMM on *A. podagraria* adaxial anthocyanins.

| ***A. podagraria* adaxial anthocyanins** |  |  |  |
| --- | --- | --- | --- |
|  | df | F | p-value |
| Treatment | 3 | 6.657 | 0.000231 |

**Table S6D**. Summary output from GAMM on *A. podagraria* adaxial anthocyanins.

| ***A. podagraria* adaxial anthocyanins** |  |  |  |  |  |
| --- | --- | --- | --- | --- | --- |
|  |  |  |  |  |  |
|  | Estimate | Std. Error | t value | Pr(>\|t\|) |  |
| (Intercept) | 0.149643 | 0.005707 | 26.222 | < 2e-16 | *** |
| TreatmentNoBlue | -0.01985 | 0.005118 | -3.878 | 0.00013 | *** |
| TreatmentNoUV | -0.02248 | 0.00545 | -4.125 | 4.84E-05 | *** |
| TreatmentNoUV<350nm | -0.01769 | 0.005967 | -2.965 | 0.00328 | ** |
|  |  |  |  |  |  |
|  | edf | Ref.df | F | p-value |  |
| s(Week):TreatmentControl | 3.22E+00 | 3.219 | 33.322 | < 2e-16 | *** |
| s(Week):TreatmentNoBlue | 2.19E+00 | 2.186 | 26.503 | 8.37E-12 | *** |
| s(Week):TreatmentNoUV | 2.99E+00 | 2.985 | 22.524 | 3.12E-13 | *** |
| s(Week):TreatmentNoUV<350nm | 3.13E+00 | 3.132 | 23.822 | 2.51E-14 | *** |
| ti(Week,Treatment) | 1.13E-07 | 12 | 0 | 0.0767 | . |
| t2(YEAR,Week,Stand,PLOT) | 4.42E+00 | 9 | 3.427 | 2.56E-06 | *** |
|  |  |  |  |  |  |
| **Comparison against UV filter** |  |  |  |  |  |
|  | Estimate | Std. Error | t value | Pr(>\|t\|) |  |
| (Intercept) | 0.130152 | 0.005546 | 23.469 | < 2e-16 | *** |
| TreatmentControl | 0.020653 | 0.005167 | 3.997 | 8.10E-05 | *** |
| TreatmentNoBlue | 0.002684 | 0.004968 | 0.54 | 0.589 |  |
| TreatmentNoUV<350nm | 0.005544 | 0.00539 | 1.029 | 0.305 |  |

**Table S6E**. ANOVA output from LME on *A. nemorosa* adaxial anthocyanins.

| ***A. nemorosa* adaxial anthocyanins** |  |  |  |  |
| --- | --- | --- | --- | --- |
|  | numDF | denDF | F-value | p-value |
| (Intercept) | 1 | 40 | 1231.783 | <.0001 |
| Week | 1 | 40 | 6.2043 | 0.017 |
| StandType | 1 | 14 | 2.7508 | 0.1194 |
| Treatment | 3 | 40 | 0.5344 | 0.6613 |
| StandType:Treatment | 3 | 40 | 1.1978 | 0.3229 |

**Table S6F**. Summary output from LME on *A. nemorosa* adaxial anthocyanins.

| ***A. nemorosa* adaxial anthocyanins** |  |  |  |  |  |
| --- | --- | --- | --- | --- | --- |
|  | Value | Std.Error | DF | t-value | p-value |
| (Intercept) | 0.3670226 | 0.077436 | 40 | 4.739662 | 0 |
| Week | -0.0067697 | 0.003307 | 40 | -2.04714 | 0.0473 |
| StandTypeEvergreen | -0.0480043 | 0.024198 | 14 | -1.98378 | 0.0672 |
| TreatmentNoBlue | -0.0325862 | 0.017299 | 40 | -1.88367 | 0.0669 |
| TreatmentNoUV | -0.0177469 | 0.017433 | 40 | -1.01801 | 0.3148 |
| TreatmentNoUV>350nm | -0.0106209 | 0.01731 | 40 | -0.61358 | 0.543 |
| StandTypeEvergreen:TreatmentNoBlue | 0.0611929 | 0.03682 | 40 | 1.661934 | 0.1043 |
| StandTypeEvergreen:TreatmentNoUV | 0.0397424 | 0.032296 | 40 | 1.23057 | 0.2257 |
| StandTypeEvergreen:TreatmentNoUV>350nm | 0.0103153 | 0.033809 | 40 | 0.305103 | 0.7619 |

**Table S6G**. ANOVA output from GAMM on *F. vesca* adaxial anthocyanins.

| ***F. vesca* adaxial anthocyanins** |  |  |  |
| --- | --- | --- | --- |
|  |  |  |  |
|  | df | F | p-value |
| StandType | 1 | 8.115 | 0.00451 |
| Treatment | 3 | 3.769 | 0.01052 |

**Table S6H.** Summary output from GAMM on *F. vesca* adaxial anthocyanins.

| ***F. vesca* adaxial anthocyanins** |  |  |  |  |  |
| --- | --- | --- | --- | --- | --- |
|  |  |  |  |  |  |
|  | Estimate | Std. Error | t value | Pr(>\|t\|) |  |
| (Intercept) | 0.1402 | 0.006683 | 20.979 | < 2e-16 | *** |
| StandTypeEvergreen | -0.02609 | 0.00916 | -2.849 | 0.00451 | ** |
| TreatmentNoBlue | 0.008418 | 0.005289 | 1.592 | 0.11187 |  |
| TreatmentNoUV | -0.00512 | 0.005122 | -0.999 | 0.3182 |  |
| TreatmentNoUV<350nm | -0.00718 | 0.005255 | -1.365 | 0.17252 |  |
| StandTypeEvergreen:TreatmentNoBlue | -0.00557 | 0.006898 | -0.808 | 0.41944 |  |
| StandTypeEvergreen:TreatmentNoUV | -0.00777 | 0.006853 | -1.134 | 0.25708 |  |
| StandTypeEvergreen:TreatmentNoUV<350nm | 0.002933 | 0.007188 | 0.408 | 0.68334 |  |
|  |  |  |  |  |  |
|  | edf | Ref.df | F | p-value |  |
| s(Week) | 6.42E+00 | 6.421 | 15.982 | <2e-16 | *** |
| ti(Week,Treatment) | 2.57E-08 | 12 | 0 | 0.478 |  |
| t2(YEAR,Week,StandType,Stand,PLOT) | 1.54E+01 | 21 | 9.494 | <2e-16 | *** |

**Table S6I**. ANOVA output from GAMM on *M. bifolium* adaxial anthocyanins.

| ***M. bifolium* adaxial anthocyanins** |  |  |  |
| --- | --- | --- | --- |
|  |  |  |  |
|  | df | F | p-value |
| Treatment | 3 | 3.933 | 0.00966 |

**Table S6J**. Summary output from GAMM on *M. bifolium* adaxial anthocyanins.

| ***M.bifolium* adaxial anthocyanins** |  |  |  |  |  |
| --- | --- | --- | --- | --- | --- |
|  |  |  |  |  |  |
|  | Estimate | Std. Error | t value | Pr(>\|t\|) |  |
| (Intercept) | 0.124143 | 0.003862 | 32.148 | <2e-16 | *** |
| TreatmentNoBlue | 0.014348 | 0.0049 | 2.928 | 0.0039 | ** |
| TreatmentNoUV | 0.000102 | 0.004222 | 0.024 | 0.9807 |  |
| TreatmentNoUV<350nm | -0.00139 | 0.004761 | -0.292 | 0.7709 |  |
|  |  |  |  |  |  |
|  | edf | Ref.df | F | p-value |  |
| s(Week) | 3.787 | 3.787 | 25.502 | < 2e-16 | *** |
| ti(Week,Treatment) | 6.006 | 12 | 1.659 | 0.0028 | ** |
| t2(YEAR,Week,PLOT) | 3.158 | 6 | 2.022 | 0.00386 | ** |
|  |  |  |  |  |  |
| **Comparison against UV filter** |  |  |  |  |  |
|  | Estimate | Std. Error | t value | Pr(>\|t\|) |  |
| (Intercept) | 1.24E-01 | 3.50E-03 | 35.491 | < 2e-16 | *** |
| TreatmentControl | 1.92E-05 | 4.17E-03 | 0.005 | 0.99633 |  |
| TreatmentNoBlue | 1.45E-02 | 4.77E-03 | 3.046 | 0.00271 | ** |
| TreatmentNoUV<350nm | -1.43E-03 | 4.58E-03 | -0.313 | 0.75491 |  |

**Table S6K**. ANOVA output from GAMM on *O. acetosella* adaxial anthocyanins.

| ***O. acetosella* adaxial anthocyanins** |  |  |  |
| --- | --- | --- | --- |
|  |  |  |  |
|  | df | F | p-value |
| StandType | 1 | 17.507 | 3.27E-05 |
| Treatment | 3 | 5.241 | 0.00141 |
| StandType:Treatment | 3 | 6.519 | 0.00024 |

**Table S6L**. Summary output from GAMM on *O. acetosella* adaxial anthocyanins.

| ***O. acetosella* adaxial anthocyanins** |  |  |  |  |  |
| --- | --- | --- | --- | --- | --- |
|  |  |  |  |  |  |
|  | Estimate | Std. Error | t value | Pr(>\|t\|) |  |
| (Intercept) | 0.177683 | 0.006398 | 27.773 | < 2e-16 | *** |
| StandTypeEvergreen | -0.03201 | 0.007651 | -4.184 | 3.27E-05 | *** |
| TreatmentNoBlue | -0.00417 | 0.006731 | -0.619 | 0.5363 |  |
| TreatmentNoUV | 0.0127 | 0.007024 | 1.808 | 0.07109 | . |
| TreatmentNoUV<350nm | 0.020448 | 0.00707 | 2.892 | 0.00396 | ** |
| StandTypeEvergreen:TreatmentNoBlue | 0.007269 | 0.007424 | 0.979 | 0.32793 |  |
| StandTypeEvergreen:TreatmentNoUV | -0.01711 | 0.007718 | -2.217 | 0.02697 | * |
| StandTypeEvergreen:TreatmentNoUV<350nm | -0.02107 | 0.007825 | -2.693 | 0.00727 | ** |
|  |  |  |  |  |  |
|  | edf | Ref.df | F | p-value |  |
| s(Week) | 7.52E+00 | 7.517 | 35.172 | < 2e-16 | *** |
| ti(Week,Treatment) | 8.65E-08 | 12 | 0 | 0.992 |  |
| t2(YEAR,Week,StandType,Stand,PLOT) | 7.70E+00 | 14 | 5.483 | 5.41E-14 | *** |
|  |  |  |  |  |  |
| **Comparison against UV filter** |  |  |  |  |  |
|  | Estimate | Std. Error | t value | Pr(>\|t\|) |  |
| (Intercept) | 0.191035 | 0.006614 | 28.884 | < 2e-16 | *** |
| StandTypeEvergreen | -0.05005 | 0.007993 | -6.261 | 7.08E-10 | *** |
| TreatmentControl | -0.01289 | 0.005578 | -2.31 | 0.021219 | * |
| TreatmentNoBlue | -0.01842 | 0.006161 | -2.99 | 0.002902 | ** |
| TreatmentNoUV<350nm | 0.003228 | 0.005797 | 0.557 | 0.577896 |  |
| StandTypeEvergreen:TreatmentControl | 0.019365 | 0.00687 | 2.819 | 0.004974 | ** |
| StandTypeEvergreen:TreatmentNoBlue | 0.025853 | 0.007362 | 3.512 | 0.000477 | *** |
| StandTypeEvergreen:TreatmentNoUV<350nm | 0.001967 | 0.007053 | 0.279 | 0.780363 |  |

**Table S6M**. ANOVA output from GAMM on *Q. robur* adaxial anthocyanins.

| ***Q. robur* adaxial anthocyanins** |  |  |  |
| --- | --- | --- | --- |
| Parametric | Terms: |  |  |
|  | df | F | p-value |
| Treatment | 3 | 3.351 | 0.023 |

**Table S6N**. Summary output from GAMM on *Q. robur* adaxial anthocyanins.

| ***Q. robur* adaxial anthocyanins** |  |  |  |  |  |
| --- | --- | --- | --- | --- | --- |
|  |  |  |  |  |  |
|  | Estimate | Std.Error | t-value | p-value |  |
| (Intercept) | 0.36354 | 0.01245 | 29.198 | <2e-16 | *** |
| TreatmentNoBlue | 0.03902 | 0.01772 | 2.203 | 0.0306 | * |
| TreatmentNoUV | 0.01667 | 0.01485 | 1.123 | 0.265 |  |
| TreatmentNoUV>350nm | 0.0299 | 0.01875 | 1.595 | 0.1148 |  |
| --- |  |  |  |  |  |
|  | edf | Ref.df | F | p-value |  |
| s(Week) | 1 | 1 | 7.743 | 0.00687 | ** |
| ti(Week,Treatment) | 9 | 9 | 0 | 1 |  |
| t2(Week,Stand,PLOT) | 4.384 | 7 | 4.364 | 3.29E-05 | *** |
|  |  |  |  |  |  |
| **Comparison against UV filter** |  |  |  |  |  |
|  | Estimate | Std. Error | t value | Pr(>\|t\|) |  |
| (Intercept) | 0.165914 | 0.013298 | 12.477 | <2e-16 | *** |
| TreatmentControl | -0.01613 | 0.018534 | -0.871 | 0.387 |  |
| TreatmentNoBlue | 0.016364 | 0.018088 | 0.905 | 0.369 |  |
| TreatmentNoUV<350nm | 0.006054 | 0.019846 | 0.305 | 0.761 |  |

**Table S6O.** ANOVA output from GAMM on *R. cassubicus* adaxial anthocyanins.

| ***R. cassubicus* adaxial anthocyanins** |  |  |  |  |
| --- | --- | --- | --- | --- |
|  |  |  |  |  |
|  | df | F | p-value | p-value |
| Treatment | 3 | 0.097 | 0.962 | <.0001 |

**Table S6P**. Summary output from GAMM on *R. cassubicus* adaxial anthocyanins.

| ***R. cassubicus* adaxial anthocyanins** |  |  |  |  |  |
| --- | --- | --- | --- | --- | --- |
|  |  |  |  |  |  |
|  | Value | Std.Error | DF | t-value | p-value |
| (Intercept) | 0.02805407 | 0.031568 | 54 | 0.888684 | 0.3781 |
| Week | 0.00420982 | 0.001205 | 54 | 3.493703 | 0.001 |
| TreatmentNoBlue | 0.01123649 | 0.048385 | 54 | 0.232229 | 0.8172 |
| TreatmentNoUV | 0.04951649 | 0.048836 | 54 | 1.013931 | 0.3151 |
| TreatmentNoUV>350nm | -0.00934154 | 0.040202 | 54 | -0.23237 | 0.8171 |
| Week:TreatmentNoBlue | -0.0004974 | 0.001821 | 54 | -0.27317 | 0.7858 |
| Week:TreatmentNoUV | -0.00234265 | 0.002 | 54 | -1.17117 | 0.2467 |
| Week:TreatmentNoUV>350nm | 0.00028692 | 0.001557 | 54 | 0.184315 | 0.8545 |
|  |  |  |  |  |  |
|  | edf | Ref.df | F | p-value |  |
| s(Week) | 2.791 | 2.791 | 13.912 | 1.29E-05 | *** |
| t2(Week,REALPLOT) | 3.407 | 5 | 3.704 | 0.00199 | ** |
|  |  |  |  |  |  |
| **Comparison against UV filter** |  |  |  |  |  |
|  | Estimate | Std. Error | t value | Pr(>\|t\|) |  |
| (Intercept) | 0.133915 | 0.006173 | 21.694 | < 2e-16 | *** |
| StandTypeEvergreen | -0.03206 | 0.008606 | -3.725 | 0.000209 | *** |
| TreatmentControl | 0.00814 | 0.004718 | 1.725 | 0.084873 | . |
| TreatmentNoBlue | 0.014653 | 0.004703 | 3.116 | 0.001903 | ** |
| TreatmentNoUV<350nm | 0.003572 | 0.004791 | 0.746 | 0.456139 |  |
| StandTypeEvergreen:TreatmentControl | 0.006999 | 0.006864 | 1.02 | 0.308218 |  |
| StandTypeEvergreen:TreatmentNoBlue | 0.001023 | 0.006893 | 0.148 | 0.882061 |  |
| StandTypeEvergreen:TreatmentNoUV<350nm | 0.004039 | 0.00719 | 0.562 | 0.574386 |  |
