# Supplementary material for: Understorey light quality affects leaf pigments and leaf phenology in different plant functional types
Source: Physiol Plant. 2022 Jun 14;174(3):e13723. doi: 10.1111/ppl.13723 (PMC9328371; doi:10.1111/ppl.13723)
Supplement: Supplementary file 2 — Appendix S2 Supporting Information [file PPL-174-0-s001.docx]

Supplemental Material 2

### **Appendix 2: Methodological considerations**

We assessed differences in the index of seasonal epidermal flavonol and anthocyanin content in our study using an optical leaf sensor. This is based on the assumption that the relationship between absorbance indices is approximately linear with their respective leaf pigment contents. A positive correlation has been reported between Dualex absorbance indices and flavonoid content for some plant species (Agati and Tattini 2010, Morales et al. 2010), including some of the understorey species in our study (Hartikainen et al., 2020). However, there is also evidence that in some alpine plant species leaf optical absorbance measured with the Dualex is does not correlated with total flavonoid content (Lefebvre et al. 2016). It is therefore possible that some of the species-specific trends we report in optically measured epidermal flavonols and anthocyanins throughout the growing season could be due to species-specific differences in the relationship between the optical methods used and the phenolic content in leaves.

In common with other studies filtering solar radiation, there can be a heating effect beneath the filters (Tosserams et al. 1996). It is for this reason that we included a transparent control filter. However, the effect of heating was very small in our study (an average 0.3°C higher in comparison to ambient understorey), and did not differ between filters. Furthermore, the flavonol and anthocyanin values fell within the normal range reported for these species in these stands and followed similar seasonal trends (Hartikainen et al. 2020). Likewise, the values for leaf senescence under the control filter were similar to those under ambient conditions (Fig. S12).

**Supplemental References**

Agati G, Tattini M. 2010. Multiple functional roles of flavonoids in photoprotection. *New Phytologist* 186: 786-793.

Hartikainen SM, Pieristè M, Lassila J, Robson TM. 2020. Seasonal patterns in spectral irradiance and leaf UV-A absorbance under forest canopies. *Frontiers in Plant Science* 10: 1762.

Lefebvre T, Millery-Vigues A, Gallet C. 2016. Does leaf optical absorbance reflect the polyphenol content of alpine plants along an elevational gradient? *Alpine Botany* 126: 177-185.

Morales LO, Tegelberg R, Brosche M, Keinänen M, Lindfors A, Aphalo PJ. 2010. Effects of solar UV-A and UV-B radiation on gene expression and phenolic accumulation in Betula pendula leaves. *Tree Physiology* 30: 923-934.

Tosserams M, de Sà AP, Rozema J. 1996. The effect of solar UV radiation on four plant species occurring in a coastal grassland vegetation in the Netherlands. *Physiologia Plantarum* 97: 731-739.
